# Efficient and selective hydrogenation of amides to alcohols and amines using a well-defined manganese–PNN pincer complex†
†Electronic supplementary information (ESI) available: General experimental procedures, additional schemes and figures, characterization data and NMR spectra are available. See DOI: 10.1039/c7sc00138j


**DOI:** 10.1039/c7sc00138j

**Published:** 2017-03-08

**Authors:** Veronica Papa, Jose R. Cabrero-Antonino, Elisabetta Alberico, Anke Spanneberg, Kathrin Junge, Henrik Junge, Matthias Beller

**Affiliations:** a Leibniz-Institut für Katalyse , e.V. Albert-Einstein Str. 29a , 18059 Rostock , Germany . Email: matthias.beller@catalysis.de; b Instituto di Chimica Biomolecolare , Consiglio Nazionale delle Ricerche , Tr. La Crucca 3 , 07100 Sassari , Italy

## Abstract

Novel well-defined NNP and PNP manganese pincer complexes have been synthetized and fully characterized. The catalyst **Mn-2** containing an imidazolyaminolphosphino ligand shows high activity and selectivity in the hydrogenation of a wide range of secondary and tertiary amides to the corresponding alcohols and amines, under relatively mild conditions. For the first time, more challenging substrates like primary aromatic amides including an actual herbicide can also be hydrogenated using this earth-abundant metal-based pincer catalyst.

## Abstract 2

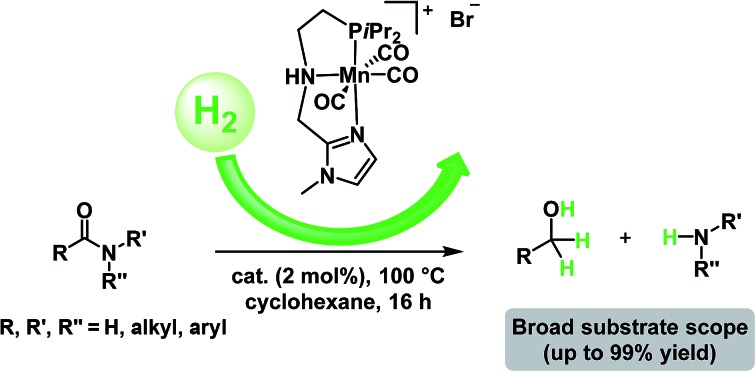
Novel manganese NNP and PNP pincer complexes have been synthesized. The active catalyst allows the efficient hydrogenation of a wide range of amides under relatively mild conditions to afford alcohols and amines in high yields.

## Introduction

Reduction of carboxylic acid derivatives represents an important process in organic synthesis with potential application in both industrial and academic settings.^1^,^2^

**Scheme 1:**
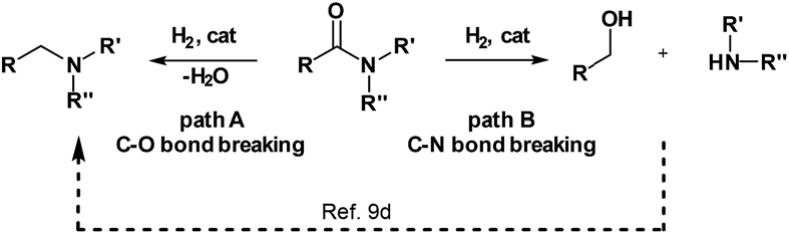
Possible reaction pathways for the hydrogenation of amides.

Traditionally, this reaction is performed using an excess of reducing agents^3^ producing stoichiometric amounts of waste-products.^4^ In contrast, catalytic hydrogenation using molecular hydrogen is a much more atom-economical and waste-free approach.4c,^5^ Unfortunately, amides constitute one of the least reactive derivatives among all classes of carbonyl compounds because of the low electrophilicity of the carbonyl group. Thus, their reduction requires harsh conditions (high pressures and temperatures) and continues to be a challenging goal.^6^ In general, the hydrogenation of amides proceeds through hemiaminal or related intermediates.5a,^7^ Here, two different pathways (C–O or C–N bond breaking, hydrogenation and hydrogenolysis, respectively) can occur (Scheme 1).5*c* The C–O cleavage pathway forms the higher amine as the product with removal of H_2_O (Scheme 1, path A), while the C–N cleavage produces the deprotected amine and the corresponding alcohol by collapse of the intermediate hemiaminal (Scheme 1, path B).

In the past decade, significant improvements in amide hydrogenation using both heterogeneous and homogeneous catalysts have been achieved. While some of the heterogeneous catalysts, *e.g.* Re–Pd/C, work under comparably mild conditions, they proceed *via* path A (Scheme 1). Notably, these precious metal systems are incompatible with aromatic and some other sensitive functional groups which can be easily reduced.^8^ No hydrogenation of aromatic rings has been observed using homogeneous ruthenium/1,1,1-tris(diphenylphosphino-methyl)ethane (triphos)-based systems in combination with specific acidic additives, as reported by Cole-Hamilton's, Leitner and Klankermayer's, Qi-Lin Zhou's groups.2b,^9^ Recently, we have demonstrated that such catalysts also reduce the amide carbonyl group to the hemiaminal, which collapses to give the alcohol and the non-alkylated amine (pathway B). Interestingly, these compounds then produce the alkylated amine *via* a hydrogen borrowing mechanism.9*d* In addition, an elegant deoxygenative hydrogenation of amides using a well-defined iridium pincer complex in the presence of a stoichiometric amount of boron Lewis acid was also reported.^10^

On the other hand, homogeneous pincer catalysts making use of metal–ligand cooperation,^11^ have shown notable activity for the hydrogenation of the amide by C–N bond cleavage (Scheme 1, path B). This transformation allows to obtain alcohols and amines from amides and is of interest as selective deprotection methodology in organic synthesis.

**Scheme 2:**
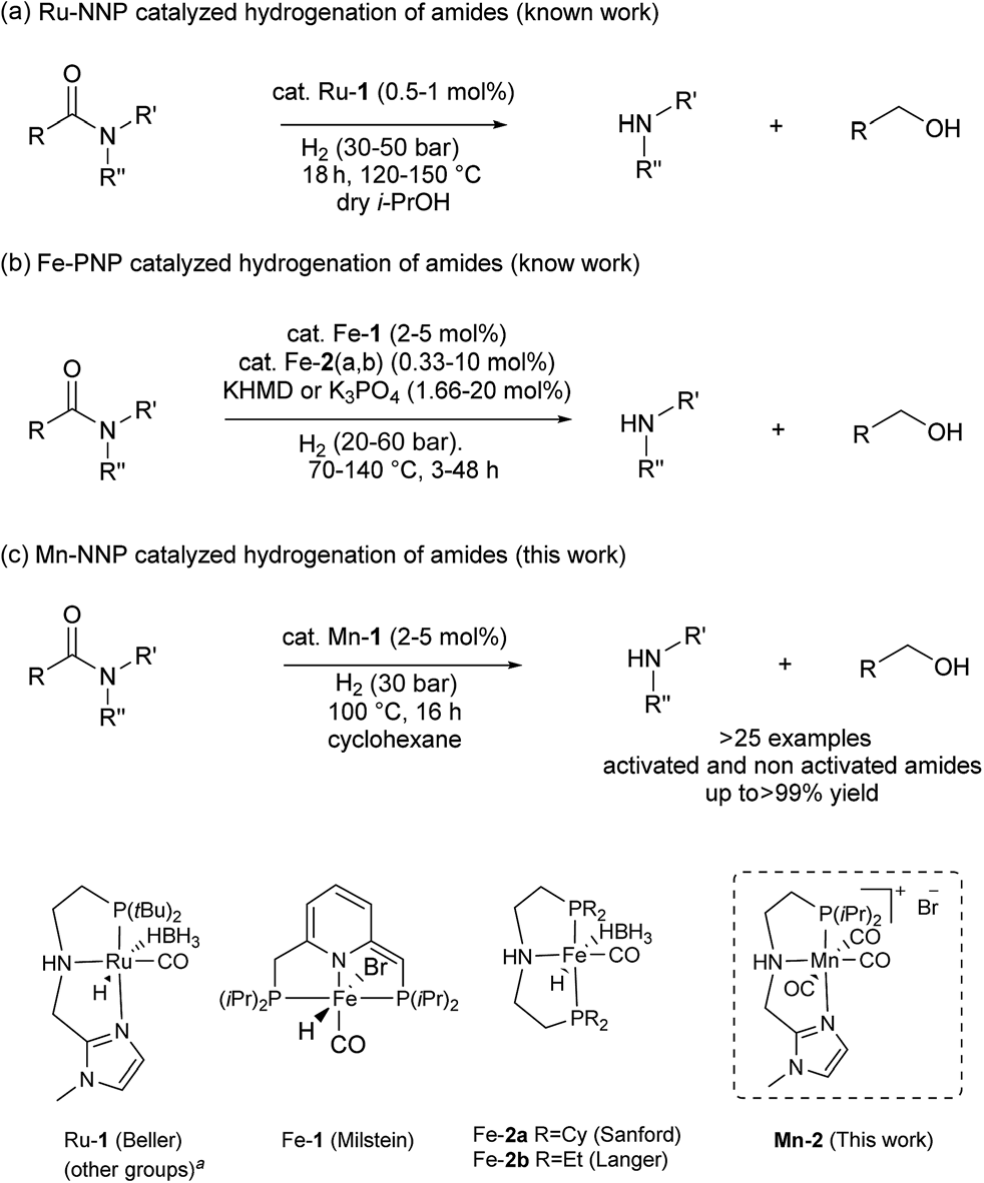
Hydrogenation of amides to alcohols and amines catalyzed by: (a) ruthenium (previous work), (b) iron (previous work) and (c) manganese (this work). ^*a*^ See ESI for other examples of Ru–pincer catalysts reported for this reaction (Scheme S1†).

The first example of this hydrogenolysis of amides to alcohols and amines was reported by Milstein using a well-defined ruthenium pincer complex.^12^ Subsequently, other examples, mainly based in ruthenium, were published by Ikariya, Saito, Bergens, Mashima, Zhang and our group (Scheme 2(a)).^13^

In general, the replacement of noble metals with inexpensive and abundant first row metals is an actual goal for catalysis as well as organometallic chemistry and attractive in terms of sustainability.^14^ Recent efforts by Milstein's, Sanford's and Langer's groups in this direction have led to the development of three homogeneous iron based catalysts for this important transformation (Scheme 2(b)).^15^ However, catalysts Fe-**1** or Fe-**2**(**a**, **b**)^15^ afforded modest conversions and yields, and/or required forcing conditions, or have a narrow scope which is limited to activated substrates like fluorine-containing amides or formamides.

In addition to iron, manganese is one of the most abundant metals in the earth crust and plays an important role in human biochemistry. In general, Mn-based complexes were employed in catalytic oxidation reactions. Moreover, special hydrosilylation and electrocatalytic reactions were reported.^16^ Very recently, the design and development of new Mn-based complex catalysts for other applications, *e.g.* reduction reactions, became very appealing.16b,^17^,^18^ In this context, here we describe the synthesis of three novel well-defined Mn–pincer complexes and their catalytic application in the efficient and selective hydrogenation of secondary and tertiary amides to the corresponding alcohols and amines under relatively mild conditions. In addition, more challenging substrates like primary benzamides could be hydrogenated in moderate yields. To the best of our knowledge, with the exception of the previously reported iron-based systems, there is no another example of this transformation catalyzed by non-noble metal-based catalytic system.

## Results and discussion

### Ligand and metal complex synthesis

**Scheme 3:**
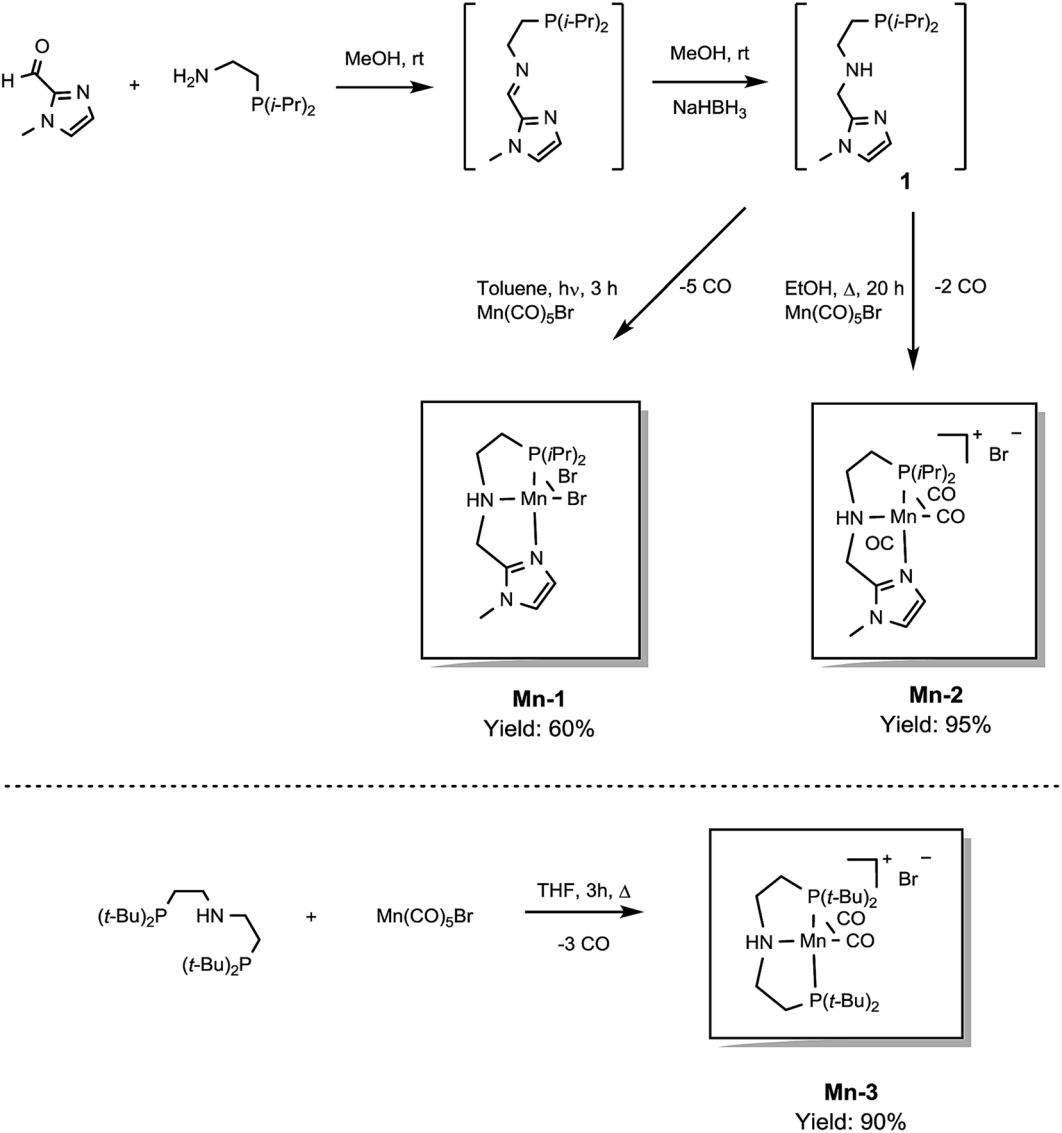
Synthesis of pincer ligand (**1**) and manganese complexes (**Mn-1**, **Mn-2** and **Mn-3**).

In previous studies, the important role of pincer ligands for bifunctional hydrogenations was clarified.^19^ While most catalysts are based on PNP ligands, other complexes which combine soft and hard donors and/or hemilabile groups have been shown to be beneficial in ester2a,^20^ and amide reduction.12*b* Following our previous work concerning the synthesis of the NNP-type imidazolylphosphine pincer ligand family and the corresponding ruthenium complexes,^21^ that were successfully applied in amide hydrogenation,13*g* we envisaged the possibility to prepare the first Mn–NNP imidazolylphosphine pincer complexes and to test their potential as reduction catalysts. For this purpose, initially the NNP pincer ligand 2-(diisopropylphosphanyl)-*N*-[(1-methyl-1*H*-imidazol-2-yl)methyl]ethan-1-amine **1** (Scheme 3), was prepared in a one-pot two-step synthesis.13g,^21^ When ligand **1** was reacted with commercially available Mn(CO)_5_Br in toluene upon irradiation with UV light for 3 hours, **Mn-1** was obtained in 60% yield (Scheme 3).^22^ To our delight the complex was crystallized by vapour diffusion of *n*-heptane into a concentrated DCM solution of the compound and the molecular structure was determined by X-ray diffraction (Fig. S1,† ESI). The molecular structure shows a distorted trigonal bipyramidal geometry in which the ligand is coordinated to the manganese in a tripodal fashion.

**Fig. 1:**
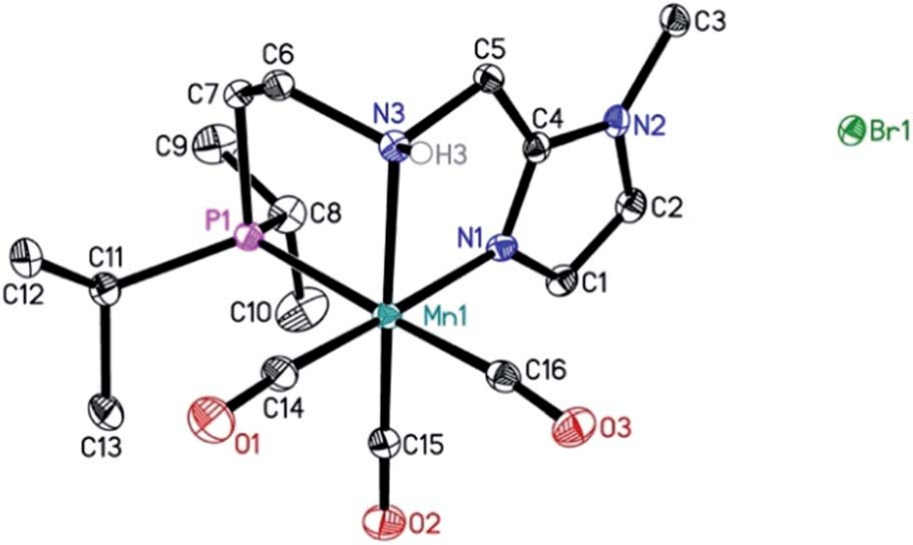
Molecular structure of manganese complex **Mn-2** with thermal ellipsoids drawn at the 30% probability level. Hydrogen atoms other than H3 have been omitted for the sake of clarity.

In addition, a Mn(i) complex was prepared by addition of the manganese precursor to ligand **1** in ethanol at 90 °C in the absence of light. In this way the tris-carbonyl ionic NNP Mn(i) complex **Mn-2** is formed in 95% yield (Scheme 3). Crystals suitable for X-ray diffraction analysis were obtained by slow evaporation of an ethanolic solution under a gentle flow of argon. Compared to complex **Mn-1** the molecular structure shown in Fig. 1 23 exhibits a distorted octahedral geometry in which the PNN ligand adopts a facial coordination, one CO ligand is *trans* to the central aliphatic nitrogen, thus acting as a tripodal ligand rather than pincer, similar to the previously reported PNP–Mn complex.18*h* The structure of **Mn-2** was further proven by NMR and IR spectroscopy, high resolution mass spectroscopy and elemental analysis (Fig. S9, see ESI†).

In general, the complex has a low molecular symmetry (*C*_1_ is the symmetry point group) and its IR spectrum shows three bands in the carbonyl region at 2027, 1943 and 1916 (partly overlapping) cm^–1^ which demonstrates the presence of three CO molecules coordinated to the metal center.

For comparative catalytic studies, in addition to the two well-defined **Mn-1** and **Mn-2** NNP pincer complexes, the synthesis of an ionic PNP Mn(i) complex **Mn-3** was also performed (the molecular structure of this complex is shown in Fig. S10, see ESI†). All the complexes synthesized are stable and do not decompose easily in the presence of air, the catalytic properties result unaltered after one month.

### Catalytic hydrogenation of amides using Mn–pincer complexes

To test the potential of the new complexes as catalysts, the hydrogenation of benzanilide **2** to afford aniline **3** and benzyl alcohol **4** was chosen as benchmark reaction.

Initially the three complexes were tested using 4 mol% of Mn catalysts under 50 bar of H_2_ and 130 °C in the presence of a slight excess of base^24^ and *t*-amyl alcohol as solvent. Under these conditions only complex **Mn-2** showed activity affording products **3** and **4** in quantitative yields (Table 1, entry 2–4). Next, variations of pressure, temperature, catalyst loading and base concentration were performed (Table 1, entry 6–13). It was found that **Mn-2** was efficient also under milder conditions (30 bar of H_2_, 120 °C, 10 mol% KO^*t*^Bu), even if the catalyst loading was reduced to 2.5 mol% (Table 1, entry 9) and the products **3** and **4** were obtained in 90% and 89% yields, respectively. However a further reduction of the amount of catalyst or base was detrimental (Table 1, entries 10–13). Notably, no reaction took place in the absence of base as predictable in a system with non-innocent pincer ligand (Table 1, entry 5). This effect of the base suggests that the active catalytic species is the amido complex, obtained by deprotonation of N–H and resulting in the release of one CO molecule. Then the proposed mechanism mights be the outersphere catalytic cycle already investigate in previous work regarding hydrogenation of carboxylic derivatives catalyzed by these type of pincer complexes.2a,13g,18g,h

**Table 1:** Hydrogenation of benzanilide **2** to aniline **3** and benzyl alcohol **4** with manganese complexes **Mn-1–3**

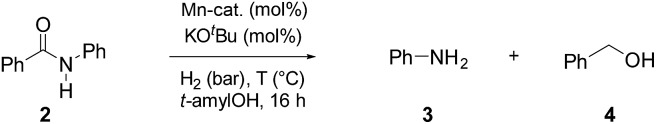							
Entry^*a*^	H_2_ (bar)	*T* (°C)	Mn-cat. (mol%)	KO^*t*^Bu (mol%)	Conv.^*b*^ (%)	**3** ^*b*^ (%)	**4** ^*b*^ (%)
1	50	130	—	15	—	—	—
2	50	130	**Mn-1** (4)	15	—	—	—
3	50	130	**Mn-2** (4)	15	>99	>99	98
4	50	130	**Mn-3** (4)	15	—	—	—
5	50	130	**Mn-2** (4)	—	—	—	—
6	50	130	**Mn-2** (4)	10	>99	97	91
7	30	130	**Mn-2** (4)	10	>99	96	93
8	30	120	**Mn-2** (4)	10	>99	>99	90
9	30	120	**Mn-2** (2.5)	10	98	90	89
10	30	120	**Mn-2** (2.5)	8	95	89	82
11	30	120	**Mn-2** (2.5)	6	72	65	62
12	30	120	**Mn-2** (2.5)	4	51	45	40
13	30	120	**Mn-2** (1.5)	8	47	32	29

^*a*^Standard reaction conditions: benzanilide **2** (49.30 mg, 0.25 mmol), Mn-cat. (2.5–4 mol%), KO^*t*^Bu (4–15 mol%), *t*-amylOH (2 mL), 30–50 bar of H_2_, 120 °C over 16 h.

^*b*^Conversion of **2** and yields of **3** and **4** were calculated by GC using hexadecane as external standard.

**Fig. 2:**
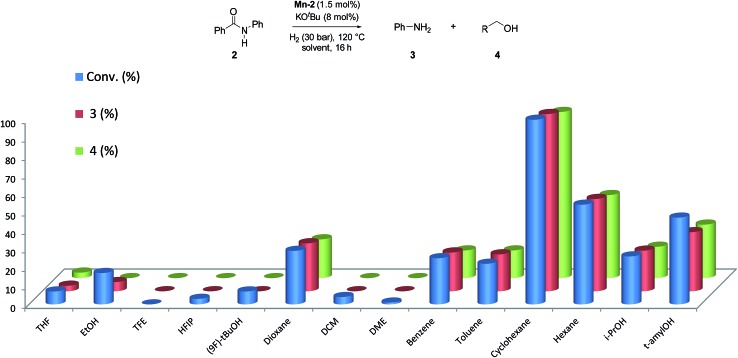
Study of the solvent effect in the hydrogenation of benzanilide **2** to aniline **3** and benzyl alcohol **4** catalyzed by **Mn-2** complex. Standard reaction conditions: benzanilide **2** (49.30 mg, 0.25 mmol), **Mn-2** (1.78 mg, 0.00375 mmol, 1.5 mol%), KO^*t*^Bu (2.13 mg, 0.019 mmol, 8 mol%), solvent (2 mL), 30 bar of H_2_, 120 °C over 16 h. Conversion of **2** and yields of **3** and **4** were calculated by GC using hexadecane as external standard.

Next, we explored the solvent effect on the catalytic activity of **Mn-2** (Fig. 2).

In order to observe positive or negative effects of the different solvents, the screening was carried out at 30 bar of H_2_ and 120 °C using 1.5 mol% **Mn-2** and 8 mol% of base. Surprisingly, among all the solvents tested (more than 13), cyclohexane was found to give the best results for this system (Fig. 2). This is a rather unexpected effect for this type of reactions and ether-, alcohol-, arene-, and fluorinated-solvents as well as other alkanes provided significantly lower activities. In this context, it is interesting to note that cyclohexane allowed for efficient transfer hydrogenation of nitriles catalyzed by a well-defined NNP Co imidazolylphosphine pincer complex.^25^

Having identified cyclohexane as the best solvent for our reaction, a fine tuning of the initially selected optimized conditions was carried out. Catalytic activity did not decrease when the temperature was reduced from 120 °C to 100 °C and the catalyst loadings from 2.5 to 2 mol% (Table 2, entry 2).

**Table 2:** Hydrogenation of benzanilide **2** to aniline **3** and benzyl alcohol **4** with manganese complex **Mn-2**: fine tuning of reaction conditions

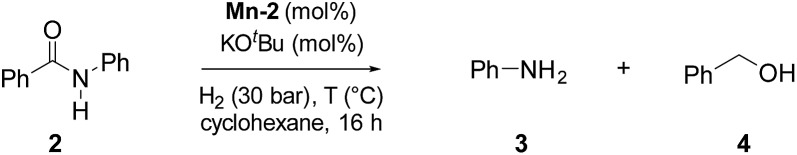						
Entry^*a*^	*T* (°C)	**Mn-2** (mol%)	KO^*t*^Bu (mol%)	Conv.^*b*^ (%)	**3** ^*b*^ (%)	**4** ^*b*^ (%)
1	120	1.5	8	>99	96	90
2	100	2	10	>99	96	90
3	100	2	8	95	88	80
4	100	2	5	29	24	24
5^*c*^	100	2	10	58	57	44
6	100	1.5	10	79	67	60
7	80	2	10	18	8	—
8^*d*^	100	2	10	—	—	—
9^*e*^	100	2	10	—	—	—
10^*f*^	100	2	10	63	53	50

^*a*^Standard reaction conditions: benzanilide **2** (49.30 mg, 0.25 mmol), **Mn-2** (1.5–2 mol%), KO^*t*^Bu (5–10 mol%), cyclohexane (2 mL), 15–30 bar of H_2_, 100–120 °C over 16 h.

^*b*^Conversion of **2** and yields of **3** and **4** were calculated by GC using hexadecane as external standard.

^*c*^Run at 15 bar of H_2_.

^*d*^Run with 2 mol% of ligand **1** in the absence of **Mn-2** complex.

^*e*^Run with 2 mol% of Mn(CO)_5_Br in the absence of **Mn-2** complex.

^*f*^Run with 2 mol% of ligand **1** and 2 mol% of Mn(CO)_5_Br.

In contrast, a substantial decrease of base loadings had a significant impact (Table 2, entries 3 and 4). Additional mitigation of the reaction temperature, hydrogen pressure and catalyst loading had negative effects on the activity (Table 2, entries 5–7). Finally, when the reaction was conducted in the presence of either ligand **1** or metal precursor no conversion was observed (Table 2, entries 8 and 9). Noticeable, *in situ* combination of Mn(CO)_5_Br and ligand **1** also afforded moderated activity in the reaction (Table 2, entry 10).

At this stage we decided to study the general applicability of the novel **Mn-2** complex in the hydrogenation of a wide range of different secondary and tertiary amides to the corresponding alcohols and amines (Table 3). Most substrates were hydrogenated in good to excellent yields under the previously selected optimized conditions at 100 °C, 30 bar of hydrogen over 16 h using cyclohexane as solvent.

**Table 3:** Substrate scope in the hydrogenation of amides to alcohols and amines catalyzed by **Mn-2** complex

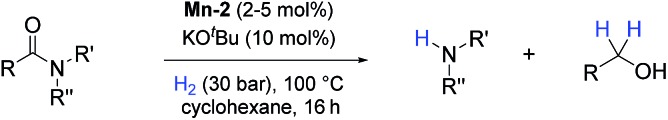					
Entry^*a*^	Amide	**Mn-2** (mol%)	Conv.^*b*^ (%)	Yield of amine^*b*^ (%)	Yield of alcohol^*b*^ (%)
1	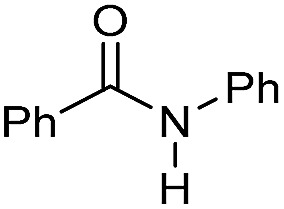	2	>99	96	90
2	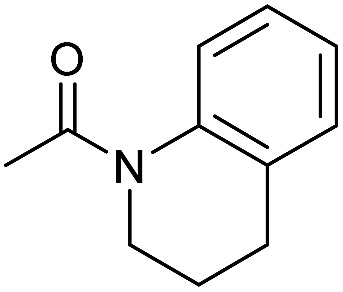	2	94	88	85
3	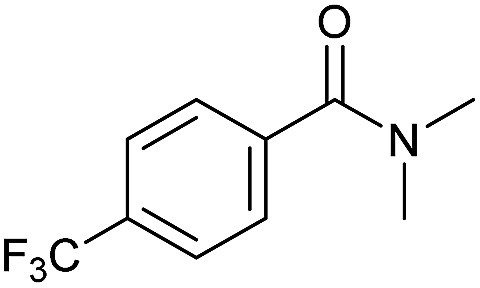	2	>99	90	n.d.
4	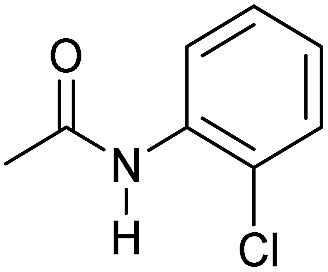	2	90	84	80
5	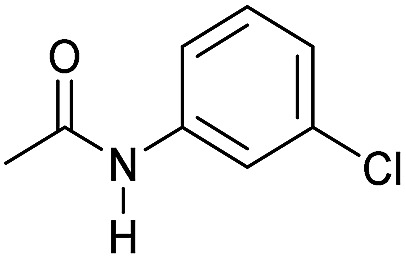	4	85	81	75
6	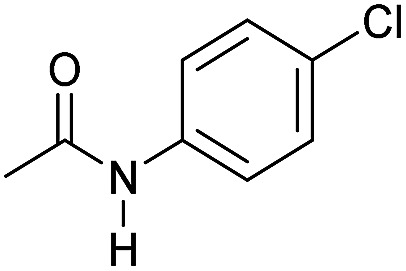	4	>99	>99	>99
7	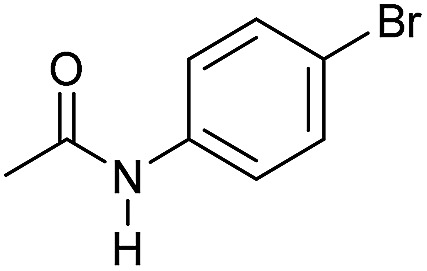	3	94	85	80
8^*c*^	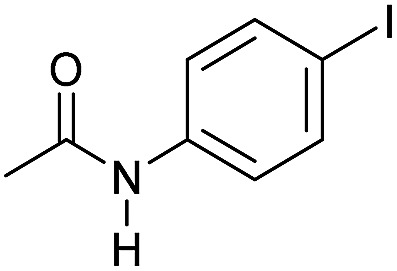	3	>99	>99	90
9	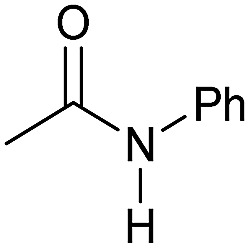	4	95	94	90
10	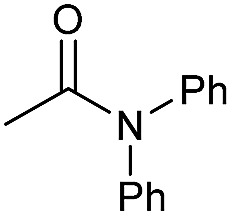	4	85	82	80
11	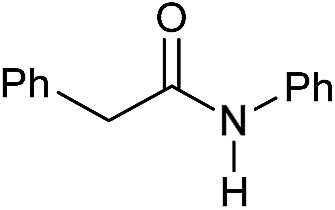	4	>99	98	94
12	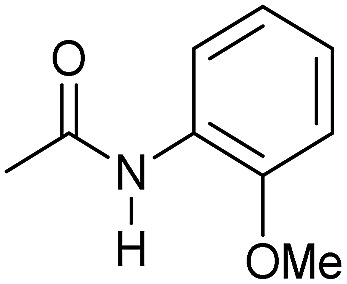	4	95	92	90
13^*c*^	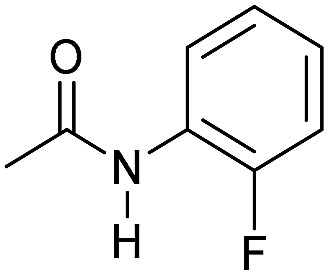	4	>99	>99	>99
14^*d*^	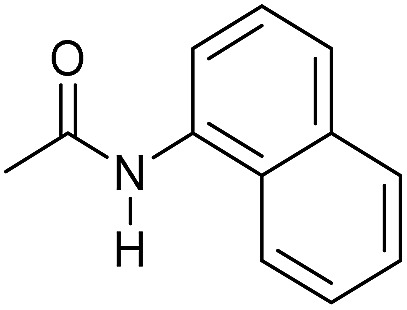	2	95	90	85
15^*c*^ ^,^^*d*^	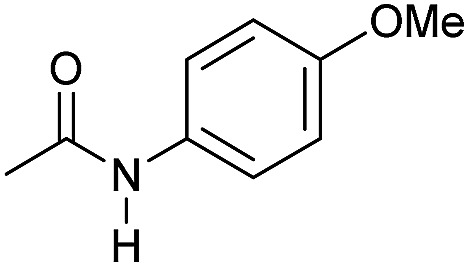	2	82	81	78
16^*d*^	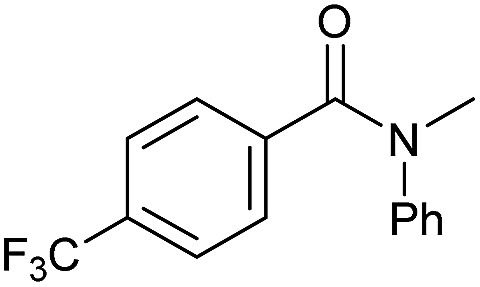	3	>99	>99	>99
17^*c*^ ^,^^*d*^	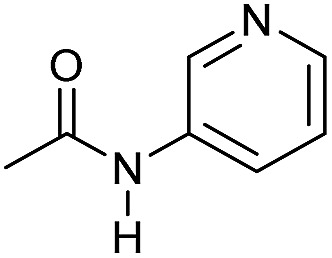	4	>99	>99	90
18^*c*^ ^,^^*d*^	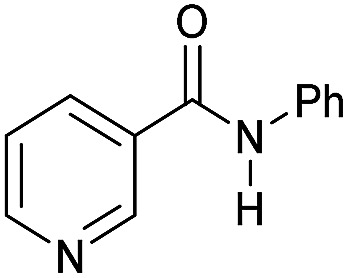	5	98	97	96

^*a*^Standard reaction conditions: amide (0.25 mmol), **Mn-2** cat. (2–5 mol%), KO^*t*^Bu (2.8 mg, 0.025 mmol, 10 mol%), cyclohexane (2 mL), 30 bar of H_2_, 100 °C over 16 h.

^*b*^Conversion of the amide and yield of the alcohol and amine were calculated by GC using hexadecane as external standard.

^*c*^The reaction was carried out using cyclohexane/*t*-amylOH (1.5/0.5) mixture as solvent (2 mL).

^*d*^Run at 120 °C.

Even less reactive electrophilic amides were converted in very good yields using up to 5 mol% catalyst loading at 100–120 °C. For instance, reduction of different fluoro- and chloro-substituted amides proceeded smoothly and no dehalogenation products were detected. Notably, also highly sensitive iodide was fully tolerated (Table 3, entries 4–8 and 13). Gratifyingly, benzyl-substituted amides (Table 3, entry 11) and amides containing electron-donating groups such as methoxy afforded very high conversions (Table 3, entries 12 and 15). Noticeably, **Mn-2** allowed the hydrogenation of *N*-acetyl-1,2,3,4-tetrahydroquinoline (Table 3, entry 2), as well as *N*-(naphthalen-1-yl)acetamide (Table 3, entry 14) and important bioactive pyridine-containing amides (Table 3, entries 17 and 18).

Tertiary amides including sterically hindered substrates were also successfully hydrogenated in the presence of this manganese complex. Thus, *N*,*N*-diphenylacetamide (Table 3, entry 10) and trifluoromethyl-activated amides (Table 3, entries 3 and 16) afforded excellent conversion and yields.

Unfortunately the reduction of aliphatic *N*-alkyl amide as *N*,*N*-dimethyloctanamide or non activated *N*-alkyl substituted benzamides, failed with this catalytic system.

**Scheme 4:**
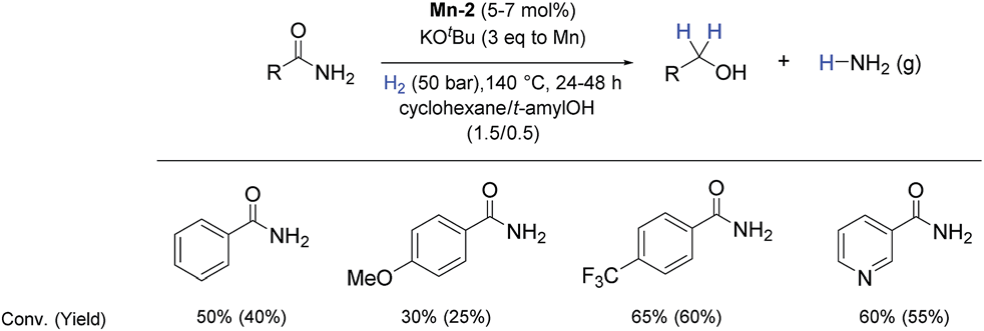
Hydrogenation of primary amides to corresponding alcohols catalyzed by **Mn-2** complex. Standard reaction conditions: amide (0.25 mmol), KO^*t*^Bu (3 eq. to Mn), cyclohexane/*t*-amylOH (1.5/0.5) mixture (2 mL), 50 bar of H_2_ and 140 °C. Specific reaction conditions for benzamide: **Mn-2** (5 mol%) over 48 h, for nicotinamide: **Mn-2** (7 mol%) over 24 h, for *p*-(trifluoromethyl)benzamide and 4-methoxybenzamide: **Mn-2** (5 mol%), over 24 h. Conversion of the amide and yields of the corresponding benzyl alcohols were calculated by GC using hexadecane as external standard.

So far, hydrogenation of primary amides – more challenging substrates – to the corresponding alcohols and ammonia has been scarcely investigated.12*b* Few examples of reduction systems are known to be active for this type of substrates such as SmI_2_-based system12*c* and the well-defined NNP ruthenium imidazolylphosphine pincer complex previously reported by us.13*g* Therefore, at this stage we explored the hydrogenation of different benzamides in the presence of **Mn-2** (Scheme 4). To our delight, benzamide and *p*-methoxybenzamide but also a heterocyclic primary amide such as nicotinamide and activated 4-(trifluoromethyl)benzamide afforded moderate to good conversion (50–65%) with high selectivity.

**Scheme 5:**
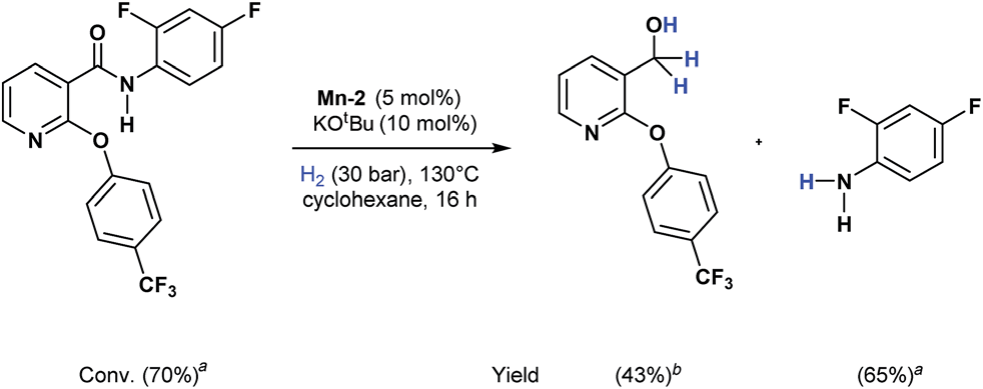
Selective hydrogenation of diflufenican to the corresponding alcohol and amine catalyzed by **Mn-2** complex. Reaction conditions: diflufenican (98.6 mg, 0.25 mmol), **Mn-2** cat. (5.92 mg, 0.0125 mmol, 5 mol%), KO^*t*^Bu (2.8 mg, 0.025 mmol, 10 mol%), cyclohexane (2 mL), 30 bar of H_2_, 130 °C over 16 h. ^*a*^Conversion of amide and yield of amine was calculated by GC using hexadecane as external standard. ^*b*^The yield was calculated by ^1^H NMR using 1,3,5-trimethoxybenzene as external standard.

Apart from aromatic and aliphatic amides also the parent formamides including alkylformamides such as *N*-octylformamide (Table 4, entry 4) or *N*-cyclohexylformamide (Table 4, entry 5) are smoothly hydrogenated under the previously optimized conditions. Even using lower catalyst loadings (1 mol% of Mn), full conversion was obtained (Table 4, entry 2). To show the applicability of our PNN pincer complex **Mn-2** finally hydrogenation of the herbicide diflufenican was investigated (Scheme 5). For the first time, this herbicide could be catalytically hydrogenated to the corresponding alcohol and amine under relatively mild conditions affording good conversion.

**Table 4:** Hydrogenation of different formamides to the corresponding amines and methanol catalyzed by **Mn-2** complex

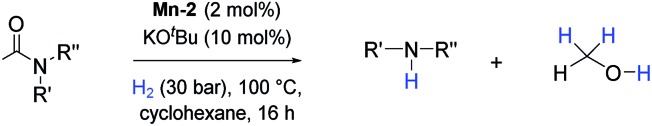				
Entry^*a*^	Formamide	Conv.^*b*^ (%)	Yield of amine^*b*^ (%)	Yield of alcohol^*b*^ (%)
1	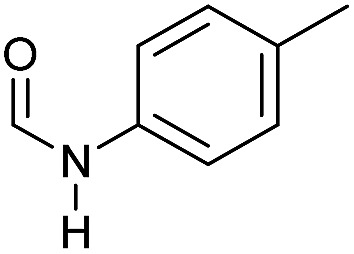	>99	95	90
2^*c*^		>99	92	88
3	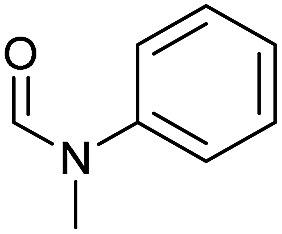	90	85	80
4	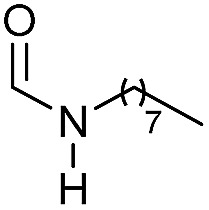	83	83	80
5	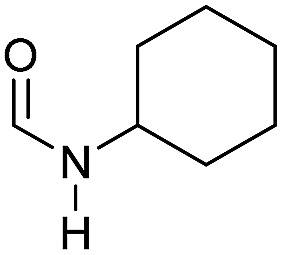	>99	>99	>99
6^*d*^	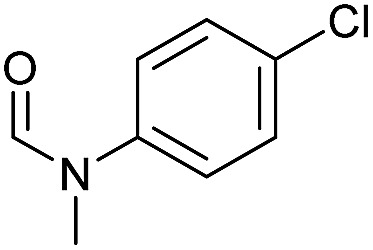	>99	>99	>99
7^*e*^	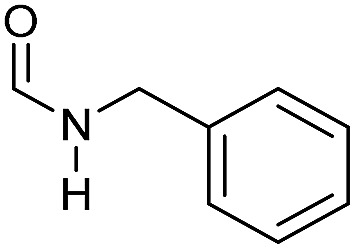	>99	>99	>99

^*a*^Standard reaction conditions: formamide (0.25 mmol), **Mn-2** cat. (2.37 mg, 0.005 mmol, 2 mol%), KO^*t*^Bu (2.8 mg, 0.025 mmol, 10 mol%), cyclohexane (2 mL), 30 bar of H_2_, 100 °C over 16 h.

^*b*^Conversion of the formamide and yield of the amine and methanol were calculated by GC using hexadecane as external standard.

^*c*^The reaction was carried out with 1 mol% of catalyst.

^*d*^The reaction was carried out with 3 mol% of catalyst.

^*e*^The reaction was carried out at 120 °C.

**Scheme 6:**
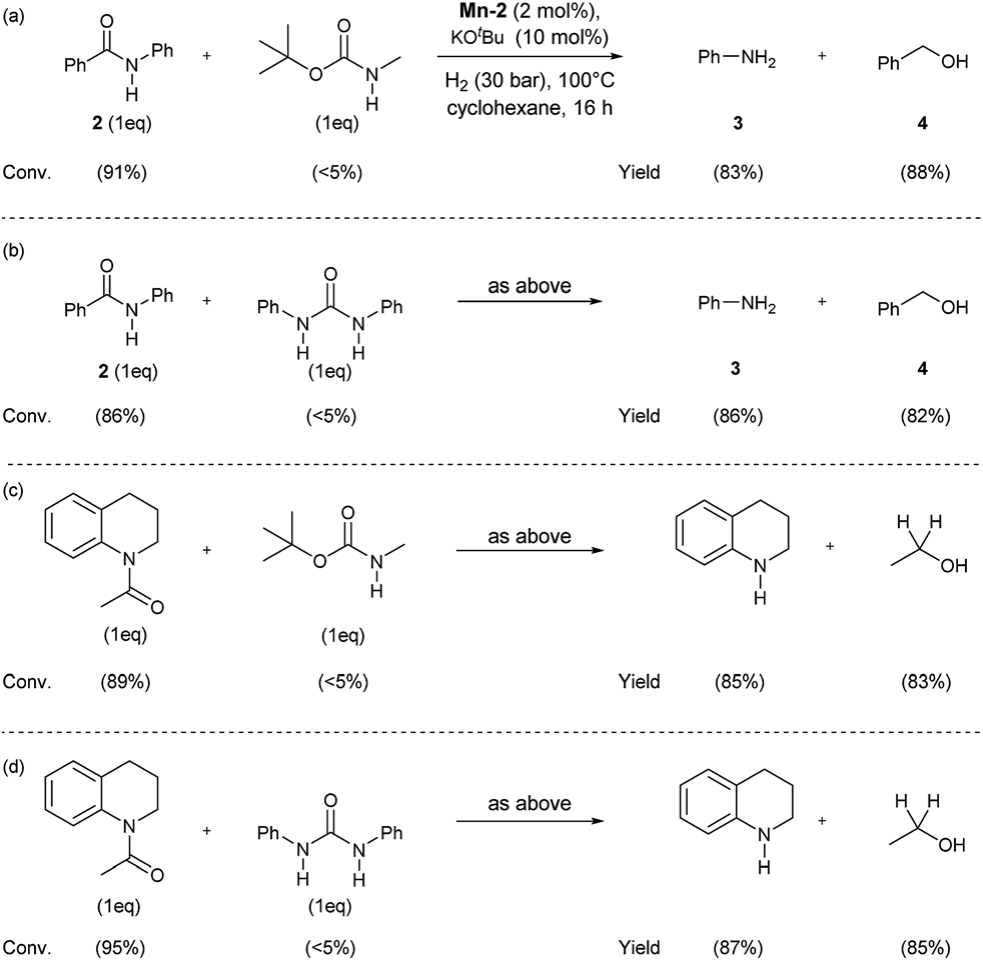
Mn-catalyzed selective hydrogenations of benzanilide **2** and *N*-acetyl-1,2,3,4-tetrahydroquinoline in the presence of *tert*-butyl-*N*-methylcarbamate (a) and (c) or diphenyl urea (b) and (d). Standard reaction conditions: substrate (0.25 mmol each one), **Mn-2** cat. (2.37 mg, 0.005 mmol, 2 mol%), KO^*t*^Bu (2.8 mg, 0.025 mmol, 10 mol%), cyclohexane (2 mL), 30 bar of H_2_, 100 °C over 16 h. Conversion of the starting material and yield of the products were calculated by GC using hexadecane as external standard.

In order to check the selectivity of the **Mn-2** pincer complex, hydrogenations of benchmark amides in the presence of additional reducible groups like carbamate or urea were carried out (Scheme 6). To our delight, using catalyst **Mn-2** highly selective reduction of benzanilide **2** and *N*-acetyl-1,2,3,4-tetrahydroquinoline to the corresponding alcohols and amines can be performed in the presence of both *tert*-butyl-*N*-methylcarbamate (Scheme 6, eqn (a) and (c)) or *N*,*N*-diphenylurea (Scheme 6, eqn (b) or (d)). It is noteworthy, that these results pave the way towards selective deprotection strategies in organic synthesis mediated by this NNP pincer complex.

## Conclusions

Different well-defined manganese–PNN pincer complexes have been synthetized for the first time. One of these complexes **Mn-2** catalyses efficiently the selective hydrogenation of a wide range of amides to the corresponding alcohols and amines under relatively mild conditions. Activated and non-activated secondary and tertiary amides and the more challenging primary amides can be hydrogenated to the corresponding products in high yields. To highlight the versatility of the protocol, the reduction of a more complex amide such as diflufenican is also included. To the best of our knowledge, this is the first example of Mn-catalyzed hydrogenation of amides. Different hydrogenation experiments have shown the excellent selectivity of the new manganese pincer complex for the hydrogenation of amides in the presence of other reducible groups. This fact opens the door to the potential application of this type of earth-abundant metal pincer complexes in organic synthetic deprotection methodologies.

## Experimental details

### General procedure for the hydrogenation of amides

A 4 mL glass vial containing a stirring bar was sequentially charged under argon with amide (0.25 mmol) and complex **Mn-2** (1–7 mol%). Afterwards, the reaction vial was capped with a septum equipped with a syringe needle and set in the alloy plate and the vial was purged with 3 cycles of vacuum and argon. Cyclohexane or cyclohexane/*t*-amyl alcohol (1.5/0.5) mixture (2 mL) and the corresponding catalytic amount of KO^*t*^Bu were sequentially added under argon and the vial was then placed into a 300 mL autoclave. Once sealed, the autoclave was purged three times with 20 bar of hydrogen, then pressurized to the desired hydrogen pressure (30–50 bar), and placed into an aluminum block that was preheated to the desired temperature (80–140 °C). After the desired reaction time of 16–48 h, the autoclave was cooled in an ice bath and the remaining gas was carefully released. Finally, *n*-hexadecane (20 mg) was added as an external standard, and the reaction mixture was diluted with ethyl acetate and analyzed by gas chromatography.

## Supplementary Material

Supplementary information
