# Supplementary material for: Efficient and selective hydrogenation of amides to alcohols and amines using a well-defined manganese–PNN pincer complex
Source: Chem Sci. 2017 Mar 8;8(5):3576–85. doi: 10.1039/c7sc00138j (PMC6092716; doi:10.1039/c7sc00138j)
Supplement: Supplementary file 1 [file SC-008-C7SC00138J-s001.pdf]

Electronic Supplementary Material (ESI) for Chemical Science.
This journal is © The Royal Society of Chemistry 2017

Electronic Supplementary Information

Efficient and Selective Hydrogenation of Amides to Alcohols and Amines using a Well-defined Manganese-PNN Pincer Complex†

Veronica Papa,[a†] Jose R. Cabrero-Antonino,[a†] Elisabetta Alberico,[a,b] Anke Spanneberg,[a] Kathrin Junge,[a] Henrik Junge,[a] and Matthias Beller[a*]

[a] Leibniz-Institut für Katalyse e.V. an der Universität Rostock, Albert-Einstein-Straße 29a, 18059 Rostock, Germany; Fax: (+49) 381-1281-5000

[b] Instituto di Chimica Biomolecolare, Consiglio Nazionale delle Ricerche, Tr. La Crucca 3, 07100 Sassari, Italy.

[*]E-mail: matthias.beller@catalysis.de

1. GENERAL INFORMATION

2. SYNTHESIS OF MANGANESE COMPLEXES Mn-1, Mn-2 and Mn-3

3. GENERAL PROCEDURE FOR THE HYDROGENATION OF AMIDES

4. ADDITIONAL SCHEME

5. REFERENCES

1. GENERAL INFORMATION

All manipulations involving air- and moisture-sensitive organometallic compounds were carried out using standard Schlenk techniques under an argon atmosphere. Dichloromethane was distilled from calcium hydride under Ar; toluene, *n*-hexane, *n*-heptane, THF, C_6H_6 and diethyl ether were distilled from sodium benzophenone ketyl under Ar; methanol and ethanol were refluxed over magnesium and distilled under Ar. Deuterated organic solvents were distilled over Na/benzophenone ketyl (THF-d_8, C_6D_6 and toluene-d_8), CaH_2 (CD_2Cl_2) or Mg (EtOH-d_6).

All other chemicals were purchased and used without further purification. All hydrogenation reactions were set up under Ar in a 300 mL autoclave (PARR Instrument Company). In order to avoid unspecific reductions, all catalytic reactions were carried out in 4 mL glass vials, which were set in an alloy plate and placed inside the autoclave. The autoclave was then purged with 30 bar of hydrogen for three times before setting the pressure to the desired value. Conversions and yields of hydrogenation reactions were determined by GC-FID, HP 6890 with FID detector, column HP530 m x 250 mm x 0.25 µm.

High resolution mass spectra were recorded on a MAT 95XP ThermoFisher Mass Spectrometer using Electrospray Ionization mode.

IR spectra were recorded on a Bruker Alpha P FT-IR spectrometer.

^{1}H NMR spectra were recorded using Bruker AV-300 (300 MHZ for 1H) and Bruker AV-400 (400 MHz for 1H) spectrometers. ^{13}C{^{1}H} NMR spectra were obtained at 75 MHz or 101 MHz. ^{31}P{1H} NMR spectra were obtained at 121 MHz or 162 MHz. NMR chemical shifts are reported in parts per million (ppm) downfield from TMS and were referenced to the residual proton resonance and the natural abundance ^{13}C resonance of the solvents. ^{31}P NMR chemical shifts are reported in parts per million downfield from H_3PO_4 and referenced to an external 85% solution of H_3PO_4. Abbreviations used in the reported NMR experiments: b, broad; s, singlet; d, doublet; t, triplet; q, quartet; m, multiplet. All measurements were carried out at room temperature unless otherwise stated.

Photolysis reactions were performed using a Lumatec Superlite 400, high pressure, Hg lamp (120W). Quartz glassware was used for all photolysis reactions.[1]

Diffraction data were collected on a Bruker Kappa APEX II Duo diffractometer using graphite monochromated Mo-K⍰ radiation. The structures were solved by direct methods (SHELXS-97[2]) and refined by full-matrix least-squares techniques on F^2 (SHELXL-2014[3]). XP (Bruker AXS) was used for molecular graphics.

Ligand 2-(diisopropylphosphanyl)-N-[(1-methyl-1H-imidazol-2-yl)methyl]ethan-1-amine (**1**) was synthetised according to a published procedure.[4]

2. SYNTHESIS OF MANGANESE COMPLEXES Mn-1, Mn-2 and Mn-3

Synthesis of {Mn(2-(diisopropylphosphanyl)-N-[(1-methyl-1H-imidazol-2-yl)methyl]ethan-1-amine}Br$_2$ (Mn-1)

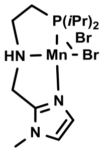

To a clear orange solution of [MnBr(CO)$_5$] (427 mg, 1.5 mmol) in 20 mL toluene was added a solution of 2-(diisopropylphosphanyl)-N-[(1-methyl-1H-imidazol-2-yl)methyl]ethan-1-amine **1** (408 mg, 1.6 mmol) in 5 mL toluene. The resulting solution was stirred at room temperature for 15 min. with no color change being observed. The solution was therefore stirred at room temperature for 3 hours under photoirradiation using a high pressure mercury lamp. During this time a yellow precipitated was formed. The suspension was concentrated and filtered. The crude product was thoroughly washed with *n*-hexane.The pale yellow solid was dried in vacuum to afford **Mn-1** (423 mg, 0.9 mmol, 60%). Colorless crystals of {Mn(2-(diisopropylphosphanyl)-N-[(1-methyl-1H-imidazol-2-yl)methyl]ethan-1-amine}Br$_2$ suitable for X-ray diffraction analysis were obtained by vapor diffusion of *n*-heptane into a concentrated DCM solution of the compound at -32°C.

Crystal data for **{Mn(2-(diisopropylphosphanyl)-N-[(1-methyl-1H-imidazol-2-yl)methyl]ethan-1-amine}Br$_2$**: $C_{13}H_{26}Br_2MnN_3P$, M = 470.10, triclinic, space group $P\bar{1}$, a = 7.6565(4), b = 8.6683(5), c = 15.6326(9) Å, α = 94.0006(14), β = 101.3485(14), γ = 111.3690(13)°, V = 935.86(9) Å^3, T = 150(2) K, Z = 2, ρ_{calcd} = 1.668 g cm^{-3}, μ(Mo Kα) = 5.055 mm^{-1}. 29925 total data, 3875 independent reflections (R_{int} = 0.0308), R_1 = 0.0439 for 3330 unique data with $I > 2\sigma(I)$ and 190 refined parameters. The final wR_2 value ($I > 2\sigma(I)$) was 0.1280. The final R values (all data) were R_1 = 0.0520 and wR_2 = 0.1356. The goodness of fit on F^2 was 1.053.

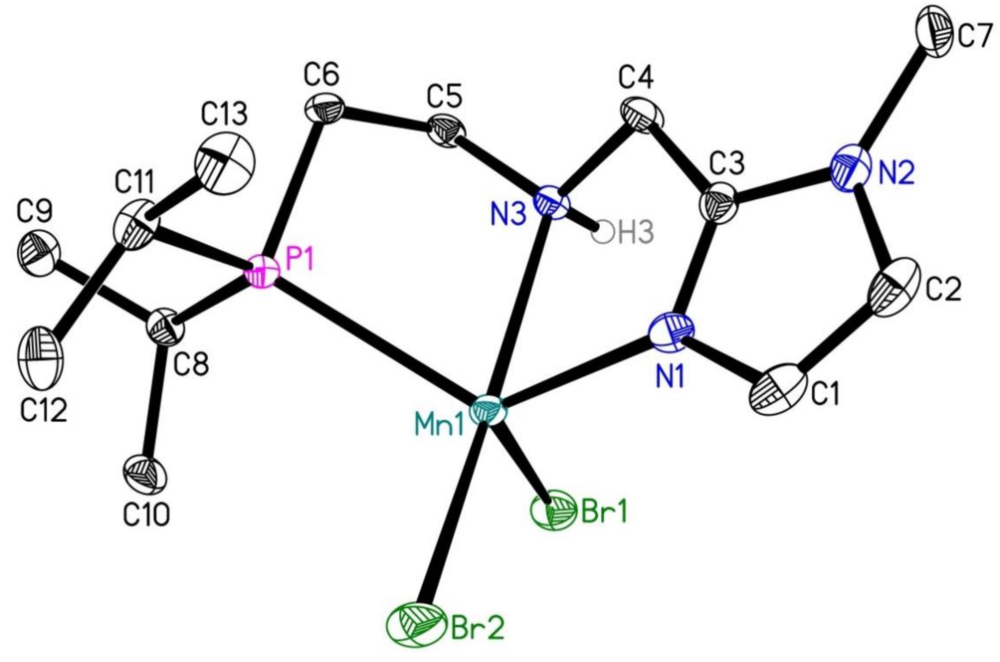

Figure S1. The molecular structure of Manganese complex **Mn-1** with thermal ellipsoids drawn at the 30% probability level. Hydrogen atoms other than H3 have been omitted for the sake of clarity.

Table S1. Bond lengths [Å] and angles [°] for **Mn-1**

C(1)-C(2)	1.516(2)
C(1)-P(1)	1.8425(15)
C(1)-H(1B)	0.9900
C(1)-H(1C)	0.9900
C(2)-N(1)	1.4898(18)
C(2)-H(2A)	0.9900
C(2)-H(2B)	0.9900
C(3)-N(1)	1.4862(18)
C(3)-C(4)	1.516(2)
C(3)-H(3A)	0.9900
C(3)-H(3B)	0.9900
C(4)-P(2)	1.8400(14)
C(4)-H(4A)	0.9900
C(4)-H(4B)	0.9900
C(5)-C(7)	1.530(2)
C(5)-C(8)	1.535(2)
C(5)-C(6)	1.538(2)
C(5)-P(1)	1.8786(15)
C(6)-H(6A)	0.9800
C(6)-H(6B)	0.9800
C(6)-H(6C)	0.9800
C(7)-H(7A)	0.9800
C(7)-H(7B)	0.9800
C(7)-H(7C)	0.9800
C(8)-H(8A)	0.9800
C(8)-H(8B)	0.9800
C(8)-H(8C)	0.9800
C(9)-C(10)	1.532(2)
C(9)-C(11)	1.536(2)
C(9)-C(12)	1.538(2)
C(9)-P(1)	1.8767(15)
C(10)-H(10A)	0.9800
C(10)-H(10B)	0.9800
C(10)-H(10C)	0.9800
C(11)-H(11A)	0.9800
C(11)-H(11B)	0.9800
C(11)-H(11C)	0.9800
C(12)-H(12A)	0.9800
C(12)-H(12B)	0.9800
C(12)-H(12C)	0.9800

C(13)-C(16)	1.535(2)
C(13)-C(15)	1.536(2)
C(13)-C(14)	1.541(2)
C(13)-P(2)	1.8803(15)
C(14)-H(14A)	0.9800
C(14)-H(14B)	0.9800
C(14)-H(14C)	0.9800
C(15)-H(15A)	0.9800
C(15)-H(15B)	0.9800
C(15)-H(15C)	0.9800
C(16)-H(16A)	0.9800
C(16)-H(16B)	0.9800
C(16)-H(16C)	0.9800
C(17)-C(19)	1.532(2)
C(17)-C(18)	1.534(2)
C(17)-C(20)	1.536(2)
C(17)-P(2)	1.8825(15)
C(18)-H(18A)	0.9800
C(18)-H(18B)	0.9800
C(18)-H(18C)	0.9800
C(19)-H(19A)	0.9800
C(19)-H(19B)	0.9800
C(19)-H(19C)	0.9800
C(20)-H(20A)	0.9800
C(20)-H(20B)	0.9800
C(20)-H(20C)	0.9800
C(21)-O(1)	1.1635(18)
C(21)-Mn(1)	1.7364(15)
C(22)-O(2)	1.1554(18)
C(22)-Mn(1)	1.7855(14)
Mn(1)-N(1)	2.0683(12)
Mn(1)-P(1)	2.3270(4)
Mn(1)-P(2)	2.3323(4)
N(1)-H(1A)	0.898(19)

C(2)-C(1)-P(1)	107.72(10)
C(2)-C(1)-H(1B)	110.2
P(1)-C(1)-H(1B)	110.2
C(2)-C(1)-H(1C)	110.2
P(1)-C(1)-H(1C)	110.2
H(1B)-C(1)-H(1C)	108.5
N(1)-C(2)-C(1)	108.24(12)
N(1)-C(2)-H(2A)	110.0

C(1)-C(2)-H(2A)	110.0
N(1)-C(2)-H(2B)	110.0
C(1)-C(2)-H(2B)	110.0
H(2A)-C(2)-H(2B)	108.4
N(1)-C(3)-C(4)	109.09(11)
N(1)-C(3)-H(3A)	109.9
C(4)-C(3)-H(3A)	109.9
N(1)-C(3)-H(3B)	109.9
C(4)-C(3)-H(3B)	109.9
H(3A)-C(3)-H(3B)	108.3
C(3)-C(4)-P(2)	107.96(10)
C(3)-C(4)-H(4A)	110.1
P(2)-C(4)-H(4A)	110.1
C(3)-C(4)-H(4B)	110.1
P(2)-C(4)-H(4B)	110.1
H(4A)-C(4)-H(4B)	108.4
C(7)-C(5)-C(8)	110.39(14)
C(7)-C(5)-C(6)	107.75(13)
C(8)-C(5)-C(6)	108.22(13)
C(7)-C(5)-P(1)	111.40(11)
C(8)-C(5)-P(1)	114.76(11)
C(6)-C(5)-P(1)	103.85(11)
C(5)-C(6)-H(6A)	109.5
C(5)-C(6)-H(6B)	109.5
H(6A)-C(6)-H(6B)	109.5
C(5)-C(6)-H(6C)	109.5
H(6A)-C(6)-H(6C)	109.5
H(6B)-C(6)-H(6C)	109.5
C(5)-C(7)-H(7A)	109.5
C(5)-C(7)-H(7B)	109.5
H(7A)-C(7)-H(7B)	109.5
C(5)-C(7)-H(7C)	109.5
H(7A)-C(7)-H(7C)	109.5
H(7B)-C(7)-H(7C)	109.5
C(5)-C(8)-H(8A)	109.5
C(5)-C(8)-H(8B)	109.5
H(8A)-C(8)-H(8B)	109.5
C(5)-C(8)-H(8C)	109.5
H(8A)-C(8)-H(8C)	109.5
H(8B)-C(8)-H(8C)	109.5
C(10)-C(9)-C(11)	109.97(14)
C(10)-C(9)-C(12)	109.09(13)
C(11)-C(9)-C(12)	107.55(13)

C(10)-C(9)-P(1)	109.36(10)
C(11)-C(9)-P(1)	112.21(11)
C(12)-C(9)-P(1)	108.60(10)
C(9)-C(10)-H(10A)	109.5
C(9)-C(10)-H(10B)	109.5
H(10A)-C(10)-H(10B)	109.5
C(9)-C(10)-H(10C)	109.5
H(10A)-C(10)-H(10C)	109.5
H(10B)-C(10)-H(10C)	109.5
C(9)-C(11)-H(11A)	109.5
C(9)-C(11)-H(11B)	109.5
H(11A)-C(11)-H(11B)	109.5
C(9)-C(11)-H(11C)	109.5
H(11A)-C(11)-H(11C)	109.5
H(11B)-C(11)-H(11C)	109.5
C(9)-C(12)-H(12A)	109.5
C(9)-C(12)-H(12B)	109.5
H(12A)-C(12)-H(12B)	109.5
C(9)-C(12)-H(12C)	109.5
H(12A)-C(12)-H(12C)	109.5
H(12B)-C(12)-H(12C)	109.5
C(16)-C(13)-C(15)	109.48(12)
C(16)-C(13)-C(14)	109.49(13)
C(15)-C(13)-C(14)	106.47(13)
C(16)-C(13)-P(2)	108.68(10)
C(15)-C(13)-P(2)	114.07(11)
C(14)-C(13)-P(2)	108.56(10)
C(13)-C(14)-H(14A)	109.5
C(13)-C(14)-H(14B)	109.5
H(14A)-C(14)-H(14B)	109.5
C(13)-C(14)-H(14C)	109.5
H(14A)-C(14)-H(14C)	109.5
H(14B)-C(14)-H(14C)	109.5
C(13)-C(15)-H(15A)	109.5
C(13)-C(15)-H(15B)	109.5
H(15A)-C(15)-H(15B)	109.5
C(13)-C(15)-H(15C)	109.5
H(15A)-C(15)-H(15C)	109.5
H(15B)-C(15)-H(15C)	109.5
C(13)-C(16)-H(16A)	109.5
C(13)-C(16)-H(16B)	109.5
H(16A)-C(16)-H(16B)	109.5
C(13)-C(16)-H(16C)	109.5

H(16A)-C(16)-H(16C)	109.5
H(16B)-C(16)-H(16C)	109.5
C(19)-C(17)-C(18)	110.05(13)
C(19)-C(17)-C(20)	108.11(13)
C(18)-C(17)-C(20)	107.82(14)
C(19)-C(17)-P(2)	111.56(11)
C(18)-C(17)-P(2)	113.53(11)
C(20)-C(17)-P(2)	105.47(10)
C(17)-C(18)-H(18A)	109.5
C(17)-C(18)-H(18B)	109.5
H(18A)-C(18)-H(18B)	109.5
C(17)-C(18)-H(18C)	109.5
H(18A)-C(18)-H(18C)	109.5
H(18B)-C(18)-H(18C)	109.5
C(17)-C(19)-H(19A)	109.5
C(17)-C(19)-H(19B)	109.5
H(19A)-C(19)-H(19B)	109.5
C(17)-C(19)-H(19C)	109.5
H(19A)-C(19)-H(19C)	109.5
H(19B)-C(19)-H(19C)	109.5
C(17)-C(20)-H(20A)	109.5
C(17)-C(20)-H(20B)	109.5
H(20A)-C(20)-H(20B)	109.5
C(17)-C(20)-H(20C)	109.5
H(20A)-C(20)-H(20C)	109.5
H(20B)-C(20)-H(20C)	109.5
O(1)-C(21)-Mn(1)	178.37(14)
O(2)-C(22)-Mn(1)	176.82(14)
C(21)-Mn(1)-C(22)	86.51(7)
C(21)-Mn(1)-N(1)	99.23(6)
C(22)-Mn(1)-N(1)	174.22(6)
C(21)-Mn(1)-P(1)	94.35(5)
C(22)-Mn(1)-P(1)	97.42(5)
N(1)-Mn(1)-P(1)	82.92(3)
C(21)-Mn(1)-P(2)	96.17(5)
C(22)-Mn(1)-P(2)	96.15(5)
N(1)-Mn(1)-P(2)	82.61(3)
P(1)-Mn(1)-P(2)	163.285(16)
C(3)-N(1)-C(2)	110.89(11)
C(3)-N(1)-Mn(1)	114.95(9)
C(2)-N(1)-Mn(1)	112.91(9)
C(3)-N(1)-H(1A)	104.2(12)
C(2)-N(1)-H(1A)	104.8(12)

Mn(1)-N(1)-H(1A)	108.2(12)
C(1)-P(1)-C(9)	105.11(7)
C(1)-P(1)-C(5)	104.67(7)
C(9)-P(1)-C(5)	111.71(7)
C(1)-P(1)-Mn(1)	100.97(5)
C(9)-P(1)-Mn(1)	121.35(5)
C(5)-P(1)-Mn(1)	110.88(5)
C(4)-P(2)-C(13)	105.86(7)
C(4)-P(2)-C(17)	103.16(7)
C(13)-P(2)-C(17)	111.27(7)
C(4)-P(2)-Mn(1)	101.51(5)
C(13)-P(2)-Mn(1)	118.66(5)
C(17)-P(2)-Mn(1)	114.19(5)

Synthesis of {Mn(CO)$_3$(2-(diisopropylphosphanyl)-N-[(1-methyl-1H-imidazol-2-yl)methyl]ethan-1-amine}Br (Mn-2)

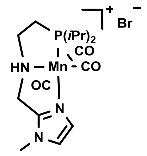

To a clear yellow solution of [MnBr(CO)$_5$] (427 mg, 1.5 mmol) in 20 mL EtOH was added a solution of 2-(diisopropylphosphanyl)-N-[(1-methyl-1H-imidazol-2-yl)methyl]ethan-1-amine **1** (408 mg, 1.6 mmol) in 5 mL EtOH. The resulting solution was stirred at room temperature for 15 min. with no color change being observed. The solution was therefore stirred at reflux temperature for 20 hours with no detectable change in the appearance of the solution being observed. Solvent was removed in vacuo and the resulting yellow solid was washed with the minimum amount of toluene, where the solid is slightly soluble, to remove unconverted [MnBr(CO)$_5$], if any, and excess ligand. Then the solid was dried under vacuum in a oil bath at 70 °C overnight. 676 mg (1.42 mmol) of Mn(CO)$_3$(2-(diisopropylphosphanyl)-N-[(1-methyl-1H-imidazol-2-yl)methyl]ethan-1-amine}Br were obtained corresponding to a 95% yield.
Yellow crystals suitable for X-ray diffraction analysis were obtained by slow evaporation of an ethanolic solution under a gentle flow of argon.

^{1}H NMR (400 MHz, Methanol-d_4, 298 K) δ 1.17 (dd, *J* = 11.7, 5.8 Hz, 3H, CH(CH$_3$)$_2$), 1.26 – 1.36 (m, *J* = 15.3, 6.3 Hz, 6H, CH(CH$_3$)$_2$, 1H, CH(CH$_3$)$_2$), 1.43 (dd, *J* = 15.4, 6.7 Hz, 3H, CH(CH$_3$)$_2$, 1H, (CH$_2$)P), 2.19 – 2.29 (bm, 1H, (CH$_2$)P), 2.52 – 2.61 (m, 1H, CH(CH$_3$)$_2$), 2.80 (m, *J* = 8.8, 4.2 Hz, 1H, HN(CH$_2$)), 3.27 (m, , 1H, HN(CH$_2$)), 3.65 (s, 3H, NCH$_3$), 3.90 (d, *J* = 17.2 Hz, 1H, Im-CH$_2$), 4.29 (d, *J* = 17.1 Hz, 1H, Im-CH$_2$), 7.00 (s, 1H, CH$_{Im}$), 7.26 (s, 1H, CH$_{Im}$).
^{1}H{^{31}P} NMR (400 MHz, Methanol-d_4, 298 K) δ 1.17 (d, *J* = 6.1 Hz, 3H, CH(CH$_3$)$_2$), 1.29 (d, *J* = 6.2 Hz, 3H, CH(CH$_3$)$_2$), 1.33 (superimposed m, *J* = 7.1 Hz, 1H, CH(CH$_3$)$_2$, 3H, CH(CH$_3$)$_2$), 1.43 (superimposed m, *J* = 6.9 Hz, 3H, CH(CH$_3$)$_2$, 1H, (CH$_2$)P), 2.24 (d, *J* = 15.4, 1H, (CH$_2$)P), 2.57 (q, *J* = 7.0 Hz, 1H, CH(CH$_3$)$_2$), 2.80 (t, *J* = 13.9, 13.4, Hz, 1H, HN(CH$_2$)), 3.29 (m, 1H, HN(CH$_2$)), 3.65 (s, 3H, NCH$_3$), 3.90 (d, *J* = 17.3 Hz, 1H, Im-CH$_2$), 4.29 (d, *J* = 17.2 Hz, 1H, Im-CH$_2$), 7.00 (s, 1H, CH$_{Im}$), 7.25 (s, 1H, CH$_{Im}$).

13C {1H} NMR (101 MHz, Methanol-d_4) δ 18.07 (d, J = 6.4 Hz, CH(<u>C</u>H$_3$)$_2$), 18.98 (s, CH(<u>C</u>H$_3$)$_2$), 20.16 (d, J = 3 Hz, CH(<u>C</u>H$_3$)$_2$), 20.85 (d, J = 2.9 Hz, CH(<u>C</u>H$_3$)$_2$), 23.46 (d, J = 17.8 Hz, <u>C</u>H$_2$P), 24.52 (d, J = 21.1 Hz, <u>C</u>H(CH$_3$)$_2$), 26.47 (d, J = 19.3 Hz, <u>C</u>H(CH$_3$)$_2$), 34.81 (bs, N<u>C</u>H$_3$), 50.24 (bs,Im<u>C</u>H$_2$), 56.61 (d, J = 9.2 Hz, N<u>C</u>H$_2$), 126.52 (s, <u>CH</u>$_{Im}$), 129.16 (s, <u>CH</u>$_{Im}$), 150.37 (s, MeN-<u>C</u>=N), CO escaped detection.

^{31}P{^{1}H} NMR (162 MHz, EtOH-d$_6$, 298 K) δ = 79.75 (s).

ATR-FTIR (solid) ῡ [cm^{-1}]: 2015 (s, ῡ CO), 1928 (s, ῡ CO), 1894 (s, ῡ CO).

ESI-HRMS (m/z, pos): Calculated for [$C_{15}H_{26}MnN_3O_2P_2$] 366.11376; found: 366.11409 [M-Br-CO]$^+$.

EA Calcd for $C_{16}H_{26}BrMnN_3O_3P_2$ (**M = 474.22 g/mo**l): C 40.52; H 5.53; N 8.86; Br 16.85; Mn 11.58; P 6.53. Found: C 40.55; H (5.62); N (9.04); Br (16.425); Mn (11.188); P (6.46).

Crystal data for **{Mn(CO)$_3$(2-(diisopropylphosphanyl)-N-[(1-methyl-1H-imidazol-2-yl)methyl]ethan-1-amine}Br**: $C_{16}H_{26}BrMnN_3O_3P$, M = 474.22, monoclinic, space group $P2_1/c$, a = 13.8967(6), b = 12.5421(5), c = 12.3081(5) Å, β = 98.3260(13)°, V = 2122.61(15) Å^3, T = 150(2) K, Z = 4, ρ_{calcd} = 1.484 g cm^{-3}, μ(Mo Kα) = 2.598 mm^{-1}. 75742 total data, 4614 independent reflections (R_{int} = 0.0456), R_1 = 0.0291 for 4284 unique data with $I > 2\sigma(I)$ and 236 refined parameters. The final wR_2 value ($I > 2\sigma(I)$) was 0.0725. The final R values (all data) were R_1 = 0.0340 and wR_2 = 0.0750. The goodness of fit on F^2 was 1.059.

The crystal under investigation was refined as a 2-component twin with 41% for the minor twin component.

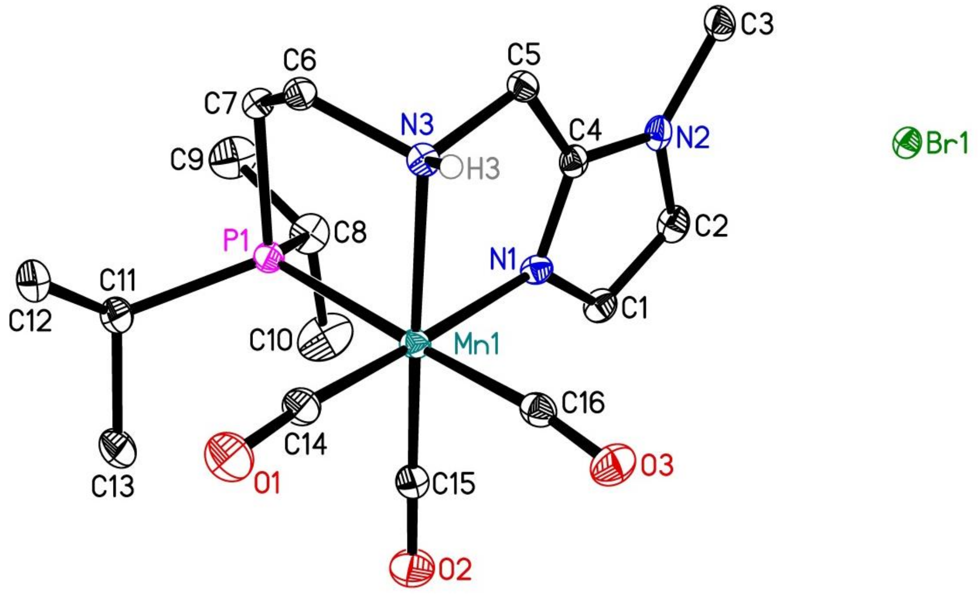

Figure S2. The molecular structure of Manganese complex **Mn-2** with thermal ellipsoids drawn at the 30% probability level. Hydrogen atoms other than H3 have been omitted for the sake of clarity.

Table S2. Bond lengths [Å] and angles [°] for **Mn-2**

C(1)-C(2)	1.361(5)
C(1)-N(1)	1.372(4)
C(1)-H(1)	0.9500
C(2)-N(2)	1.376(4)
C(2)-H(2)	0.9500
C(3)-N(2)	1.463(4)
C(3)-H(3A)	0.9800
C(3)-H(3B)	0.9800
C(3)-H(3C)	0.9800

C(4)-N(1)	1.330(4)
C(4)-N(2)	1.344(4)
C(4)-C(5)	1.477(4)
C(5)-N(3)	1.496(4)
C(5)-H(5A)	0.9900
C(5)-H(5B)	0.9900
C(6)-N(3)	1.486(4)
C(6)-C(7)	1.517(4)
C(6)-H(6A)	0.9900
C(6)-H(6B)	0.9900
C(7)-P(1)	1.834(3)
C(7)-H(7A)	0.9900
C(7)-H(7B)	0.9900
C(8)-C(10)	1.525(5)
C(8)-C(9)	1.526(5)
C(8)-P(1)	1.842(3)
C(8)-H(8)	1.0000
C(9)-H(9A)	0.9800
C(9)-H(9B)	0.9800
C(9)-H(9C)	0.9800
C(10)-H(10A)	0.9800
C(10)-H(10B)	0.9800
C(10)-H(10C)	0.9800
C(11)-C(12)	1.526(5)
C(11)-C(13)	1.536(5)
C(11)-P(1)	1.853(3)
C(11)-H(11)	1.0000
C(12)-H(12A)	0.9800
C(12)-H(12B)	0.9800
C(12)-H(12C)	0.9800
C(13)-H(13A)	0.9800
C(13)-H(13B)	0.9800
C(13)-H(13C)	0.9800
C(14)-O(1)	1.146(4)
C(14)-Mn(1)	1.798(3)
C(15)-O(2)	1.154(4)
C(15)-Mn(1)	1.789(3)
C(16)-O(3)	1.146(4)
C(16)-Mn(1)	1.831(3)
Mn(1)-N(1)	2.030(2)
Mn(1)-N(3)	2.129(2)
Mn(1)-P(1)	2.3434(9)
N(3)-H(3)	0.96(4)

C(2)-C(1)-N(1)	109.2(3)
C(2)-C(1)-H(1)	125.4
N(1)-C(1)-H(1)	125.4
C(1)-C(2)-N(2)	106.1(3)
C(1)-C(2)-H(2)	126.9
N(2)-C(2)-H(2)	126.9
N(2)-C(3)-H(3A)	109.5
N(2)-C(3)-H(3B)	109.5
H(3A)-C(3)-H(3B)	109.5
N(2)-C(3)-H(3C)	109.5
H(3A)-C(3)-H(3C)	109.5
H(3B)-C(3)-H(3C)	109.5
N(1)-C(4)-N(2)	110.3(3)
N(1)-C(4)-C(5)	121.7(3)
N(2)-C(4)-C(5)	127.9(3)
C(4)-C(5)-N(3)	108.9(2)
C(4)-C(5)-H(5A)	109.9
N(3)-C(5)-H(5A)	109.9
C(4)-C(5)-H(5B)	109.9
N(3)-C(5)-H(5B)	109.9
H(5A)-C(5)-H(5B)	108.3
N(3)-C(6)-C(7)	110.0(2)
N(3)-C(6)-H(6A)	109.7
C(7)-C(6)-H(6A)	109.7
N(3)-C(6)-H(6B)	109.7
C(7)-C(6)-H(6B)	109.7
H(6A)-C(6)-H(6B)	108.2
C(6)-C(7)-P(1)	108.8(2)
C(6)-C(7)-H(7A)	109.9
P(1)-C(7)-H(7A)	109.9
C(6)-C(7)-H(7B)	109.9
P(1)-C(7)-H(7B)	109.9
H(7A)-C(7)-H(7B)	108.3
C(10)-C(8)-C(9)	110.9(3)
C(10)-C(8)-P(1)	113.8(3)
C(9)-C(8)-P(1)	113.0(3)
C(10)-C(8)-H(8)	106.1
C(9)-C(8)-H(8)	106.1
P(1)-C(8)-H(8)	106.1
C(8)-C(9)-H(9A)	109.5
C(8)-C(9)-H(9B)	109.5
H(9A)-C(9)-H(9B)	109.5

C(8)-C(9)-H(9C)	109.5
H(9A)-C(9)-H(9C)	109.5
H(9B)-C(9)-H(9C)	109.5
C(8)-C(10)-H(10A)	109.5
C(8)-C(10)-H(10B)	109.5
H(10A)-C(10)-H(10B)	109.5
C(8)-C(10)-H(10C)	109.5
H(10A)-C(10)-H(10C)	109.5
H(10B)-C(10)-H(10C)	109.5
C(12)-C(11)-C(13)	110.0(3)
C(12)-C(11)-P(1)	113.1(2)
C(13)-C(11)-P(1)	111.0(2)
C(12)-C(11)-H(11)	107.5
C(13)-C(11)-H(11)	107.5
P(1)-C(11)-H(11)	107.5
C(11)-C(12)-H(12A)	109.5
C(11)-C(12)-H(12B)	109.5
H(12A)-C(12)-H(12B)	109.5
C(11)-C(12)-H(12C)	109.5
H(12A)-C(12)-H(12C)	109.5
H(12B)-C(12)-H(12C)	109.5
C(11)-C(13)-H(13A)	109.5
C(11)-C(13)-H(13B)	109.5
H(13A)-C(13)-H(13B)	109.5
C(11)-C(13)-H(13C)	109.5
H(13A)-C(13)-H(13C)	109.5
H(13B)-C(13)-H(13C)	109.5
O(1)-C(14)-Mn(1)	175.2(3)
O(2)-C(15)-Mn(1)	178.1(3)
O(3)-C(16)-Mn(1)	173.1(3)
C(15)-Mn(1)-C(14)	90.01(15)
C(15)-Mn(1)-C(16)	88.85(13)
C(14)-Mn(1)-C(16)	86.36(14)
C(15)-Mn(1)-N(1)	94.57(12)
C(14)-Mn(1)-N(1)	174.94(13)
C(16)-Mn(1)-N(1)	91.56(12)
C(15)-Mn(1)-N(3)	173.70(12)
C(14)-Mn(1)-N(3)	95.54(13)
C(16)-Mn(1)-N(3)	94.47(11)
N(1)-Mn(1)-N(3)	80.01(10)
C(15)-Mn(1)-P(1)	94.58(10)
C(14)-Mn(1)-P(1)	92.78(11)
C(16)-Mn(1)-P(1)	176.47(10)

N(1)-Mn(1)-P(1)	89.01(7)
N(3)-Mn(1)-P(1)	82.20(7)
C(4)-N(1)-C(1)	106.5(3)
C(4)-N(1)-Mn(1)	115.7(2)
C(1)-N(1)-Mn(1)	137.1(2)
C(4)-N(2)-C(2)	107.8(3)
C(4)-N(2)-C(3)	126.1(3)
C(2)-N(2)-C(3)	126.1(3)
C(6)-N(3)-C(5)	111.3(2)
C(6)-N(3)-Mn(1)	112.79(17)
C(5)-N(3)-Mn(1)	113.07(18)
C(6)-N(3)-H(3)	110(2)
C(5)-N(3)-H(3)	104(2)
Mn(1)-N(3)-H(3)	105(2)
C(7)-P(1)-C(8)	101.70(15)
C(7)-P(1)-C(11)	106.34(15)
C(8)-P(1)-C(11)	106.64(16)
C(7)-P(1)-Mn(1)	102.05(10)
C(8)-P(1)-Mn(1)	117.57(12)
C(11)-P(1)-Mn(1)	120.16(12)

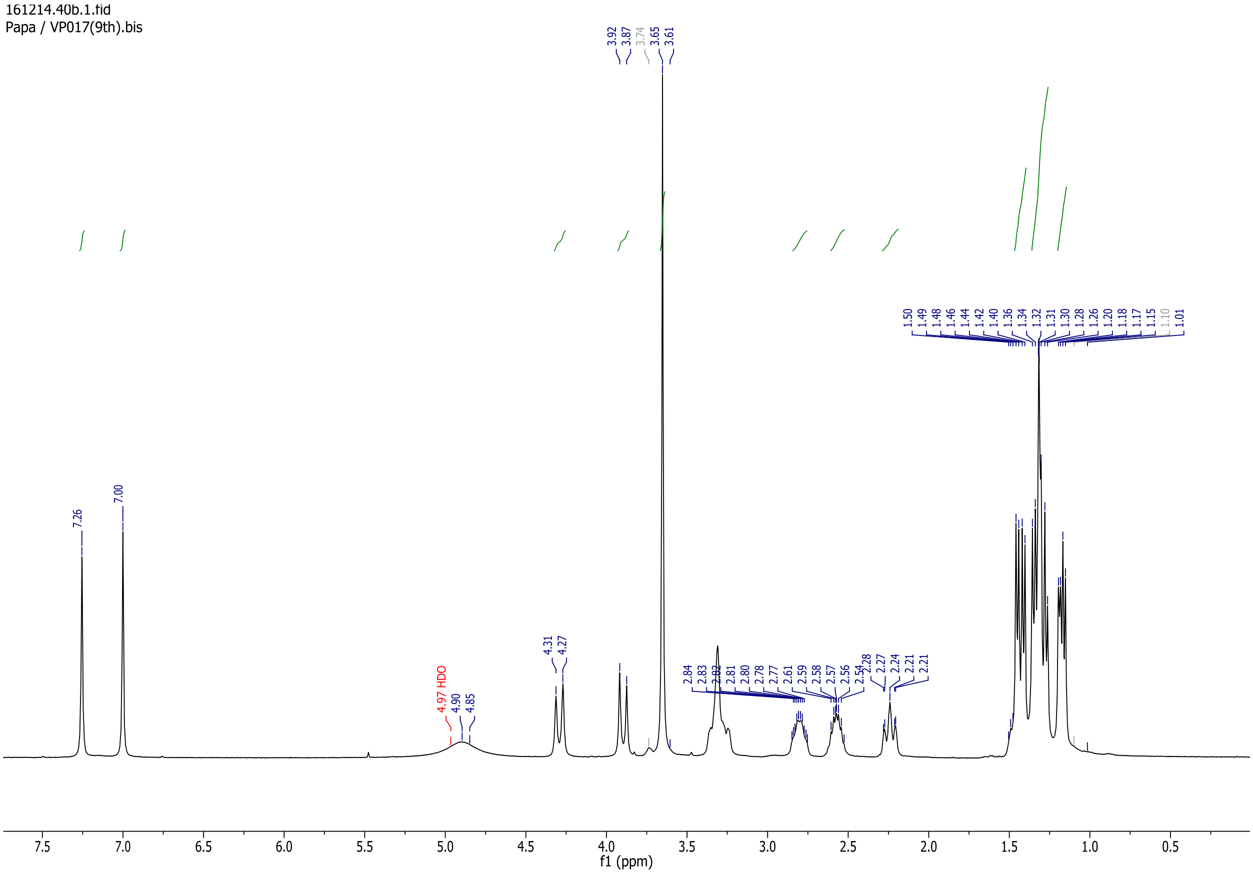

Figure S3. ^{1}H NMR (400 MHz, Methanol-d_4, 298 K) of **Mn-2**

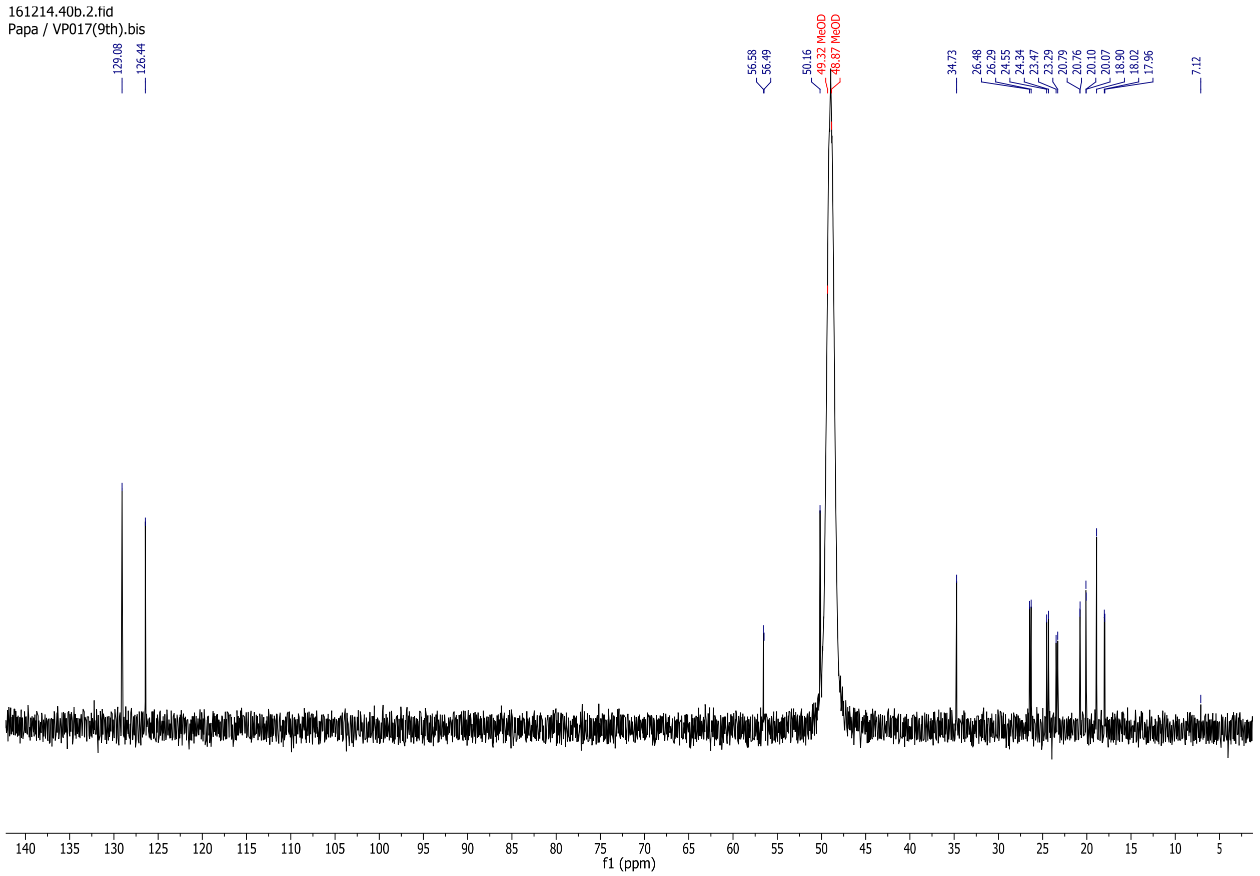

Figure S4. ^{13}C NMR (400 MHz, Methanol-d_4, 298 K) of **Mn-2**

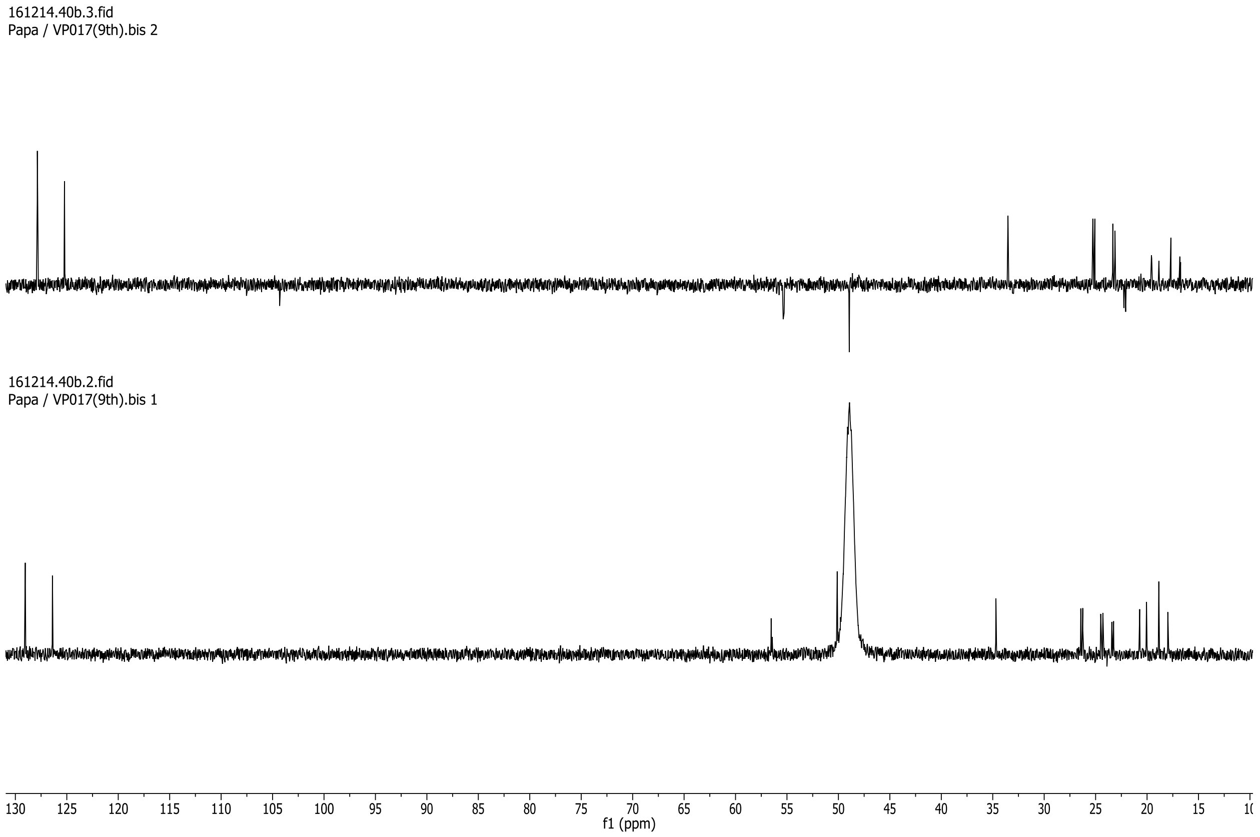

Figure S5. ^{13}C NMR (bottom spectrum) and DEPT (upper spectrum) (400 MHz, Methanol-d_4, 298 K) of **Mn-2**

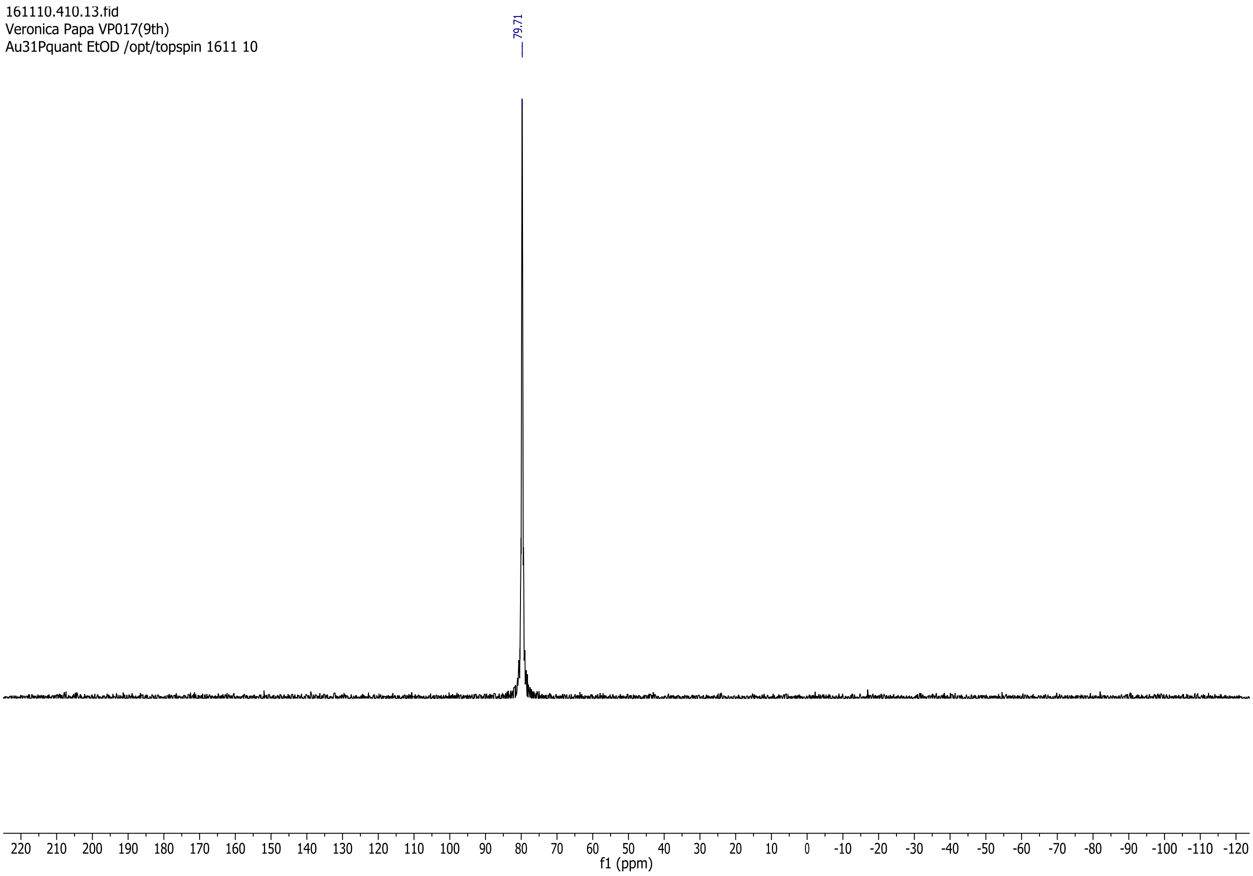

Figure S6. ^{31}P NMR (400 MHz, Ethanol-d_6, 298 K) of **Mn-2**

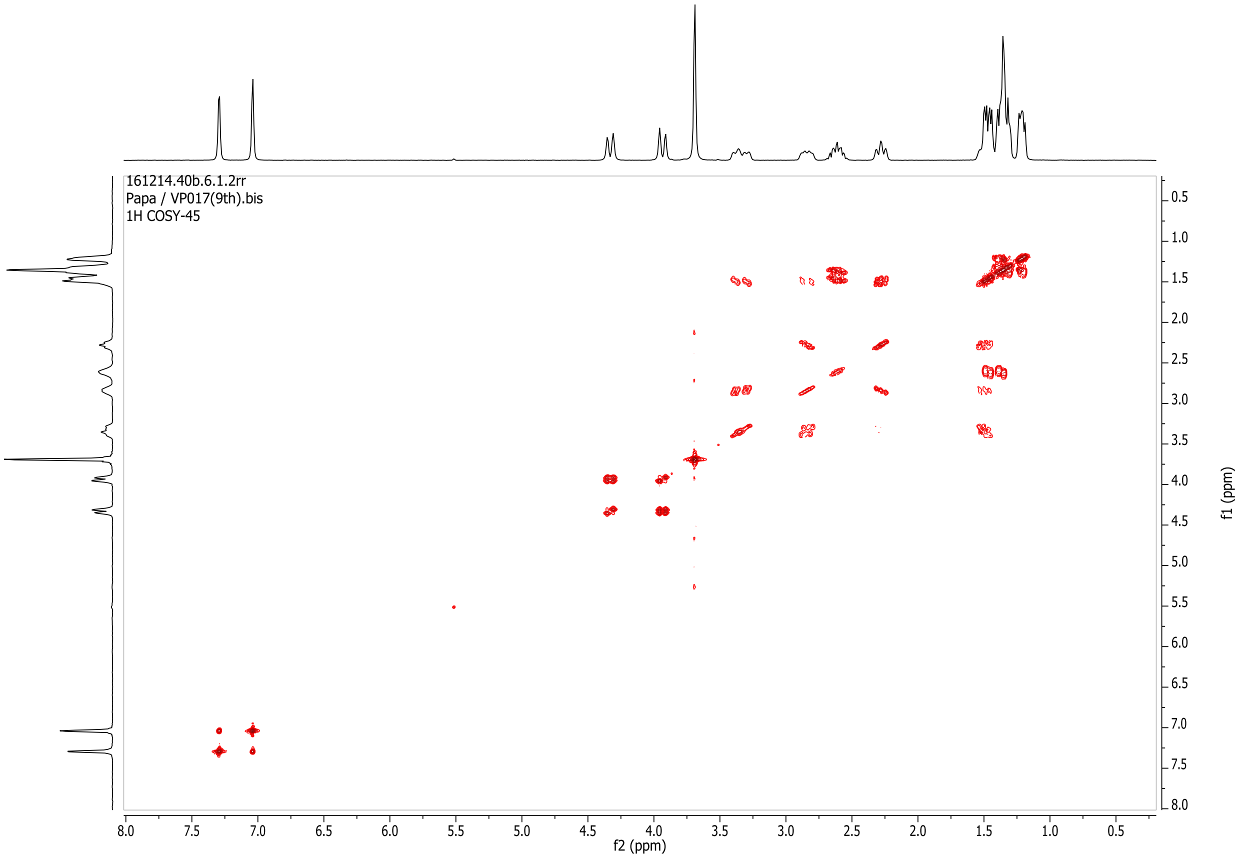

Figure S7. COSY NMR (400 MHz, Methanol-d_4, 298 K) of **Mn-2**

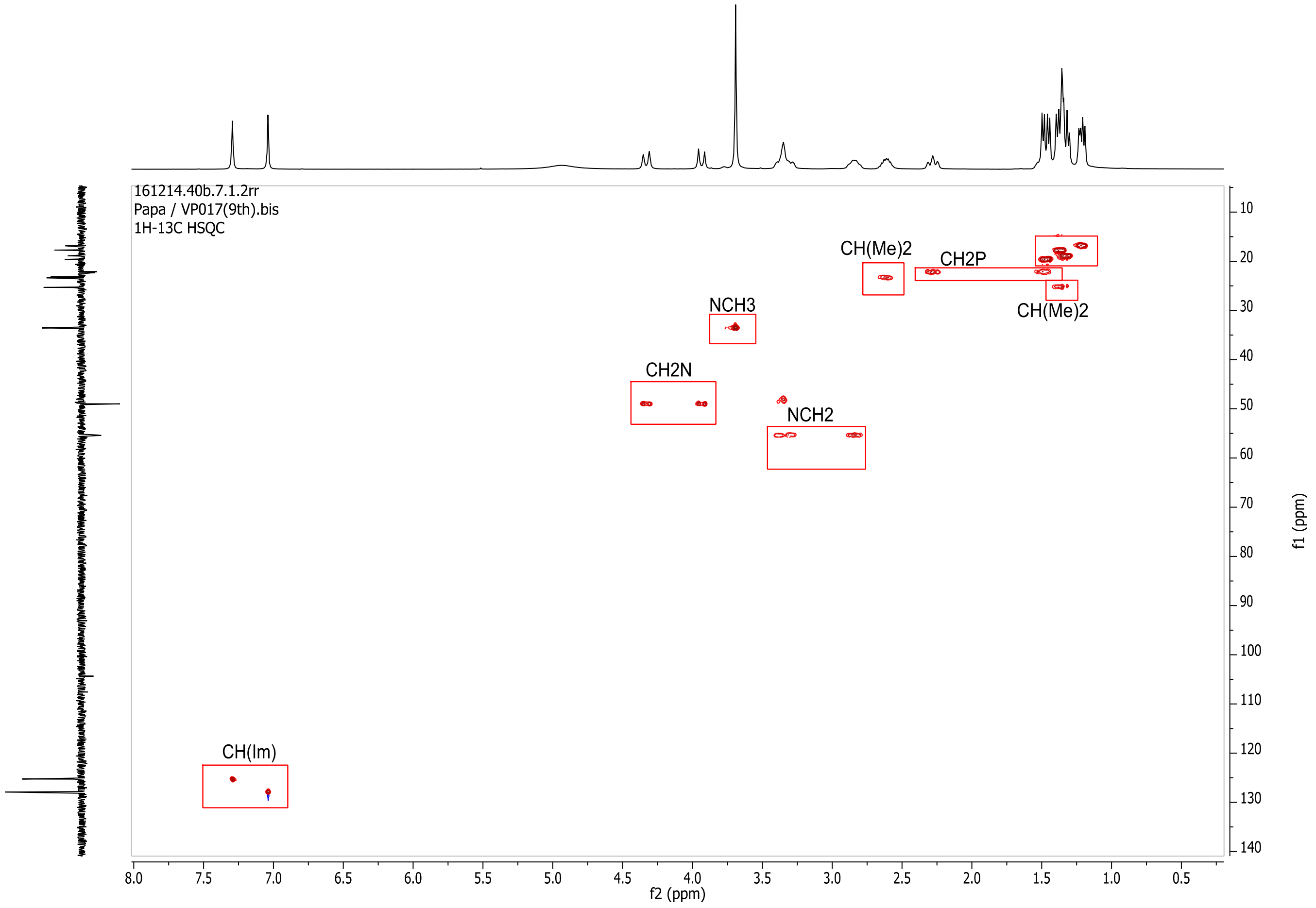

Figure S8. HSQC NMR (400 MHz, Methanol-d_4, 298 K) of **Mn-2**

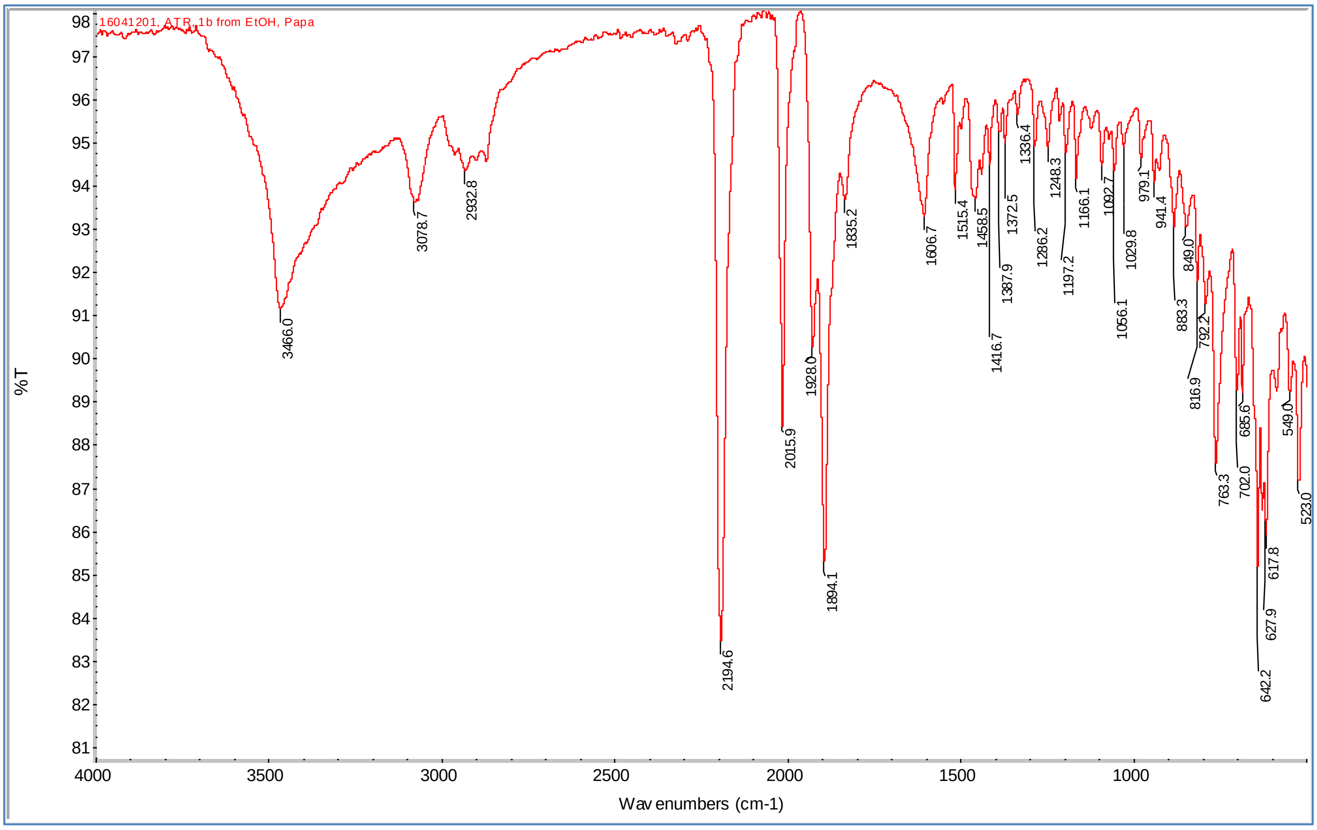

Figure S9. IR-ATR spectrum **Mn-2**.

Synthesis of {Mn(CO)$_2$(HN[(CH$_2$CH$_2$P(C(CH$_3$)$_3$]$_2$}Br (Mn-3)

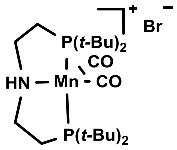

To a clear orange solution of [MnBr(CO)$_5$] (231 mg, 0.84 mmol, 1 eq) in 16 mL THF was added a solution of [HN(CH$_2$CH$_2$P(C(CH$_3$)$_3$)$_2$] (364 mg, 1 mmol, 1.2 eq) in 4 mL THF. The resulting solution was stirred at room temperature for 15 min. with no color change being observed. The solution was therefore stirred at reflux temperature for 2.5 hours during which a dark violet precipitate was formed. The suspension was concentrated and filtered. The solid was washed with the minimum amount of THF to remove unconverted [MnBr(CO)$_5$], if any, and excess ligand, followed by Et$_2$O and *n*-heptane, where the solid is not at all soluble. 417 mg of {Mn(CO)$_2$(HN[(CH$_2$CH$_2$P(C(CH$_3$)$_3$]$_2$}Br were obtained corresponding to a 90% yield. Dark violet crystals suitable for X-ray diffraction analysis were obtained by slow diffusion of Et$_2$O into a concentrated DCM solution of the compound.

^{1}H NMR (400 MHz, CD$_2$Cl$_2$, 298 K) δ = 1.11 ("d", 13.6 Hz, 18 H, PC (C<u>H</u>$_3$)$_3$), 1.39 ("d", 13.5 Hz, 18 H, PC(C<u>H</u>$_3$)$_3$), 2.38-2.47 (bm, 4H, PCH$_2$), 3.07 (bs, 2H, NCH$_2$), 4.07 (bd, J_{HP} = 19.8 Hz, 2H, NCH$_2$), 6.93 (bt, 1H, $^3J_{HH} \approx$ 10 Hz, NH);
^{1}H{^{31}P} NMR (400 MHz, CD$_2$Cl$_2$, 298 K): δ = 1.11 (s, 18 H, PC(C<u>H</u>$_3$)$_3$), 1.39 (s, 18 H, PC(C<u>H</u>$_3$)$_3$), 2.38-2.47 (bm, 4H, PCH$_2$), 3.07 (bs, 2H, NCH$_2$), 4.07 (bs, 2H, NCH$_2$), 6.93 (bt, 1H, $^3J_{HH} \approx$ 10 Hz, NH);
^{13}C{^{1}H} NMR (100 MHz, CD$_2$Cl$_2$,, 298 K) δ = 23.24 ("t", 6.8 Hz, PCH$_2$), 28.86 (bs, CH$_3$), 29.10 (bs, CH$_3$), 37.11 ("t", 8.1 Hz, <u>C</u>(CH$_3$)$_3$), 37.17 ("t", 7.3 Hz, <u>C</u>(CH$_3$)$_3$), 55.01 ("t", 5.0 Hz, NCH$_2$), CO escaped detection;
^{31}P{^{1}H} NMR (162 MHz, CD$_2$Cl$_2$, 298 K) δ = 102.78 (s).
ATR-FTIR: ῡ [cm^{-1}] 1841 (s, ν CO), 1924 (s, ν CO).
ESI-HRMS (m/z, pos): Calculated for [C22H45MnNO2P2] 472.23005; found: 472.23001 [M-Br]$^+$.
Crystal data for **{Mn(CO)$_2$(HN[(CH$_2$CH$_2$P(C(CH$_3$)$_3$]$_2$}Br**: $C_{22}H_{45}BrMnNO_2P_2$, *M* = 552.38, monoclinic, space group *P*2/*c*, *a* = 13.6682(3), *b* = 13.0374(3), *c* = 15.3870(3) Å, β = 100.0382(8)°, *V* = 2699.96(10) Å^3, *T* = 150(2) K, *Z* = 4, ρ_{calcd} = 1.359 g cm^{-3}, μ(Mo Kα) = 2.105 mm^{-1}. 35160 total data, 6209 independent reflections (R_{int} = 0.0201), R_1 = 0.0231 for 5609 unique data with $I > 2\sigma(I)$ and 278 refined parameters. The final wR_2 value ($I > 2\sigma(I)$) was 0.0573. The final *R* values (all data) were R_1 = 0.0270 and wR_2 = 0.0597. The goodness of fit on F^2 was 1.028.

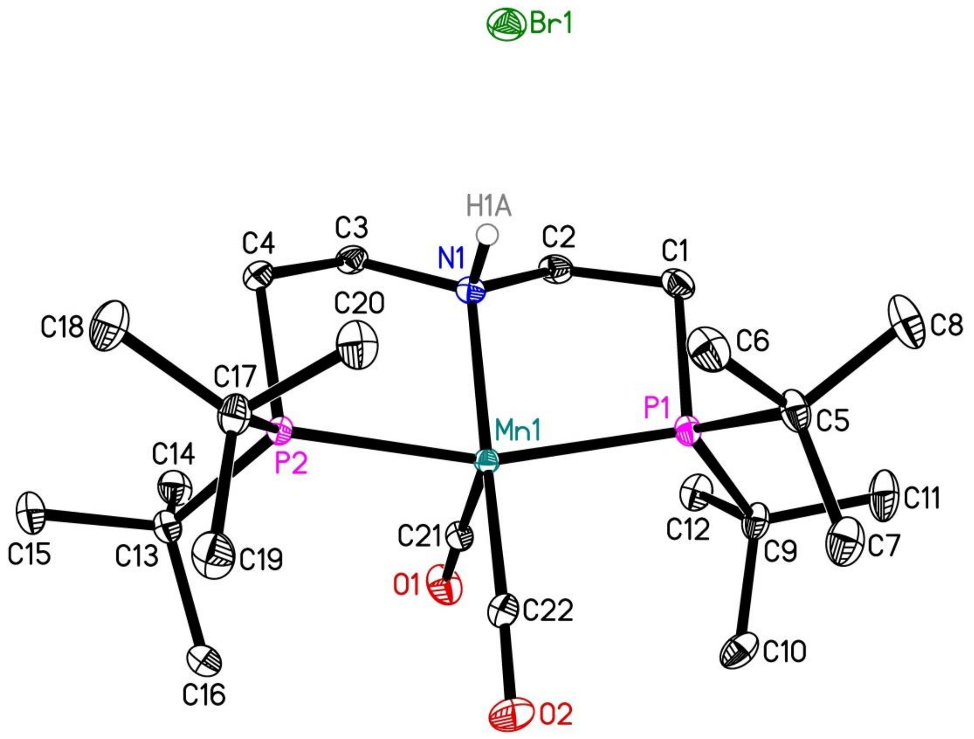

Figure S10. The molecular structure of Manganese complex **Mn-3** with thermal ellipsoids drawn at the 30% probability level. Hydrogen atoms other than H3 have been omitted for the sake of clarity.

Table S3. Bond lengths [Å] and angles [°] for **Mn-3**

C(1)-C(2)	1.516(2)
C(1)-P(1)	1.8425(15)
C(1)-H(1B)	0.9900
C(1)-H(1C)	0.9900
C(2)-N(1)	1.4898(18)
C(2)-H(2A)	0.9900
C(2)-H(2B)	0.9900
C(3)-N(1)	1.4862(18)
C(3)-C(4)	1.516(2)
C(3)-H(3A)	0.9900
C(3)-H(3B)	0.9900
C(4)-P(2)	1.8400(14)
C(4)-H(4A)	0.9900
C(4)-H(4B)	0.9900
C(5)-C(7)	1.530(2)
C(5)-C(8)	1.535(2)
C(5)-C(6)	1.538(2)
C(5)-P(1)	1.8786(15)
C(6)-H(6A)	0.9800
C(6)-H(6B)	0.9800

C(6)-H(6C)	0.9800
C(7)-H(7A)	0.9800
C(7)-H(7B)	0.9800
C(7)-H(7C)	0.9800
C(8)-H(8A)	0.9800
C(8)-H(8B)	0.9800
C(8)-H(8C)	0.9800
C(9)-C(10)	1.532(2)
C(9)-C(11)	1.536(2)
C(9)-C(12)	1.538(2)
C(9)-P(1)	1.8767(15)
C(10)-H(10A)	0.9800
C(10)-H(10B)	0.9800
C(10)-H(10C)	0.9800
C(11)-H(11A)	0.9800
C(11)-H(11B)	0.9800
C(11)-H(11C)	0.9800
C(12)-H(12A)	0.9800
C(12)-H(12B)	0.9800
C(12)-H(12C)	0.9800
C(13)-C(16)	1.535(2)
C(13)-C(15)	1.536(2)
C(13)-C(14)	1.541(2)
C(13)-P(2)	1.8803(15)
C(14)-H(14A)	0.9800
C(14)-H(14B)	0.9800
C(14)-H(14C)	0.9800
C(15)-H(15A)	0.9800
C(15)-H(15B)	0.9800
C(15)-H(15C)	0.9800
C(16)-H(16A)	0.9800
C(16)-H(16B)	0.9800
C(16)-H(16C)	0.9800
C(17)-C(19)	1.532(2)
C(17)-C(18)	1.534(2)
C(17)-C(20)	1.536(2)
C(17)-P(2)	1.8825(15)
C(18)-H(18A)	0.9800
C(18)-H(18B)	0.9800
C(18)-H(18C)	0.9800
C(19)-H(19A)	0.9800
C(19)-H(19B)	0.9800
C(19)-H(19C)	0.9800

C(20)-H(20A)	0.9800
C(20)-H(20B)	0.9800
C(20)-H(20C)	0.9800
C(21)-O(1)	1.1635(18)
C(21)-Mn(1)	1.7364(15)
C(22)-O(2)	1.1554(18)
C(22)-Mn(1)	1.7855(14)
Mn(1)-N(1)	2.0683(12)
Mn(1)-P(1)	2.3270(4)
Mn(1)-P(2)	2.3323(4)
N(1)-H(1A)	0.898(19)

C(2)-C(1)-P(1)	107.72(10)
C(2)-C(1)-H(1B)	110.2
P(1)-C(1)-H(1B)	110.2
C(2)-C(1)-H(1C)	110.2
P(1)-C(1)-H(1C)	110.2
H(1B)-C(1)-H(1C)	108.5
N(1)-C(2)-C(1)	108.24(12)
N(1)-C(2)-H(2A)	110.0
C(1)-C(2)-H(2A)	110.0
N(1)-C(2)-H(2B)	110.0
C(1)-C(2)-H(2B)	110.0
H(2A)-C(2)-H(2B)	108.4
N(1)-C(3)-C(4)	109.09(11)
N(1)-C(3)-H(3A)	109.9
C(4)-C(3)-H(3A)	109.9
N(1)-C(3)-H(3B)	109.9
C(4)-C(3)-H(3B)	109.9
H(3A)-C(3)-H(3B)	108.3
C(3)-C(4)-P(2)	107.96(10)
C(3)-C(4)-H(4A)	110.1
P(2)-C(4)-H(4A)	110.1
C(3)-C(4)-H(4B)	110.1
P(2)-C(4)-H(4B)	110.1
H(4A)-C(4)-H(4B)	108.4
C(7)-C(5)-C(8)	110.39(14)
C(7)-C(5)-C(6)	107.75(13)
C(8)-C(5)-C(6)	108.22(13)
C(7)-C(5)-P(1)	111.40(11)
C(8)-C(5)-P(1)	114.76(11)
C(6)-C(5)-P(1)	103.85(11)
C(5)-C(6)-H(6A)	109.5

C(5)-C(6)-H(6B)	109.5
H(6A)-C(6)-H(6B)	109.5
C(5)-C(6)-H(6C)	109.5
H(6A)-C(6)-H(6C)	109.5
H(6B)-C(6)-H(6C)	109.5
C(5)-C(7)-H(7A)	109.5
C(5)-C(7)-H(7B)	109.5
H(7A)-C(7)-H(7B)	109.5
C(5)-C(7)-H(7C)	109.5
H(7A)-C(7)-H(7C)	109.5
H(7B)-C(7)-H(7C)	109.5
C(5)-C(8)-H(8A)	109.5
C(5)-C(8)-H(8B)	109.5
H(8A)-C(8)-H(8B)	109.5
C(5)-C(8)-H(8C)	109.5
H(8A)-C(8)-H(8C)	109.5
H(8B)-C(8)-H(8C)	109.5
C(10)-C(9)-C(11)	109.97(14)
C(10)-C(9)-C(12)	109.09(13)
C(11)-C(9)-C(12)	107.55(13)
C(10)-C(9)-P(1)	109.36(10)
C(11)-C(9)-P(1)	112.21(11)
C(12)-C(9)-P(1)	108.60(10)
C(9)-C(10)-H(10A)	109.5
C(9)-C(10)-H(10B)	109.5
H(10A)-C(10)-H(10B)	109.5
C(9)-C(10)-H(10C)	109.5
H(10A)-C(10)-H(10C)	109.5
H(10B)-C(10)-H(10C)	109.5
C(9)-C(11)-H(11A)	109.5
C(9)-C(11)-H(11B)	109.5
H(11A)-C(11)-H(11B)	109.5
C(9)-C(11)-H(11C)	109.5
H(11A)-C(11)-H(11C)	109.5
H(11B)-C(11)-H(11C)	109.5
C(9)-C(12)-H(12A)	109.5
C(9)-C(12)-H(12B)	109.5
H(12A)-C(12)-H(12B)	109.5
C(9)-C(12)-H(12C)	109.5
H(12A)-C(12)-H(12C)	109.5
H(12B)-C(12)-H(12C)	109.5
C(16)-C(13)-C(15)	109.48(12)
C(16)-C(13)-C(14)	109.49(13)

C(15)-C(13)-C(14)	106.47(13)
C(16)-C(13)-P(2)	108.68(10)
C(15)-C(13)-P(2)	114.07(11)
C(14)-C(13)-P(2)	108.56(10)
C(13)-C(14)-H(14A)	109.5
C(13)-C(14)-H(14B)	109.5
H(14A)-C(14)-H(14B)	109.5
C(13)-C(14)-H(14C)	109.5
H(14A)-C(14)-H(14C)	109.5
H(14B)-C(14)-H(14C)	109.5
C(13)-C(15)-H(15A)	109.5
C(13)-C(15)-H(15B)	109.5
H(15A)-C(15)-H(15B)	109.5
C(13)-C(15)-H(15C)	109.5
H(15A)-C(15)-H(15C)	109.5
H(15B)-C(15)-H(15C)	109.5
C(13)-C(16)-H(16A)	109.5
C(13)-C(16)-H(16B)	109.5
H(16A)-C(16)-H(16B)	109.5
C(13)-C(16)-H(16C)	109.5
H(16A)-C(16)-H(16C)	109.5
H(16B)-C(16)-H(16C)	109.5
C(19)-C(17)-C(18)	110.05(13)
C(19)-C(17)-C(20)	108.11(13)
C(18)-C(17)-C(20)	107.82(14)
C(19)-C(17)-P(2)	111.56(11)
C(18)-C(17)-P(2)	113.53(11)
C(20)-C(17)-P(2)	105.47(10)
C(17)-C(18)-H(18A)	109.5
C(17)-C(18)-H(18B)	109.5
H(18A)-C(18)-H(18B)	109.5
C(17)-C(18)-H(18C)	109.5
H(18A)-C(18)-H(18C)	109.5
H(18B)-C(18)-H(18C)	109.5
C(17)-C(19)-H(19A)	109.5
C(17)-C(19)-H(19B)	109.5
H(19A)-C(19)-H(19B)	109.5
C(17)-C(19)-H(19C)	109.5
H(19A)-C(19)-H(19C)	109.5
H(19B)-C(19)-H(19C)	109.5
C(17)-C(20)-H(20A)	109.5
C(17)-C(20)-H(20B)	109.5
H(20A)-C(20)-H(20B)	109.5

C(17)-C(20)-H(20C)	109.5
H(20A)-C(20)-H(20C)	109.5
H(20B)-C(20)-H(20C)	109.5
O(1)-C(21)-Mn(1)	178.37(14)
O(2)-C(22)-Mn(1)	176.82(14)
C(21)-Mn(1)-C(22)	86.51(7)
C(21)-Mn(1)-N(1)	99.23(6)
C(22)-Mn(1)-N(1)	174.22(6)
C(21)-Mn(1)-P(1)	94.35(5)
C(22)-Mn(1)-P(1)	97.42(5)
N(1)-Mn(1)-P(1)	82.92(3)
C(21)-Mn(1)-P(2)	96.17(5)
C(22)-Mn(1)-P(2)	96.15(5)
N(1)-Mn(1)-P(2)	82.61(3)
P(1)-Mn(1)-P(2)	163.285(16)
C(3)-N(1)-C(2)	110.89(11)
C(3)-N(1)-Mn(1)	114.95(9)
C(2)-N(1)-Mn(1)	112.91(9)
C(3)-N(1)-H(1A)	104.2(12)
C(2)-N(1)-H(1A)	104.8(12)
Mn(1)-N(1)-H(1A)	108.2(12)
C(1)-P(1)-C(9)	105.11(7)
C(1)-P(1)-C(5)	104.67(7)
C(9)-P(1)-C(5)	111.71(7)
C(1)-P(1)-Mn(1)	100.97(5)
C(9)-P(1)-Mn(1)	121.35(5)
C(5)-P(1)-Mn(1)	110.88(5)
C(4)-P(2)-C(13)	105.86(7)
C(4)-P(2)-C(17)	103.16(7)
C(13)-P(2)-C(17)	111.27(7)
C(4)-P(2)-Mn(1)	101.51(5)
C(13)-P(2)-Mn(1)	118.66(5)
C(17)-P(2)-Mn(1)	114.19(5)

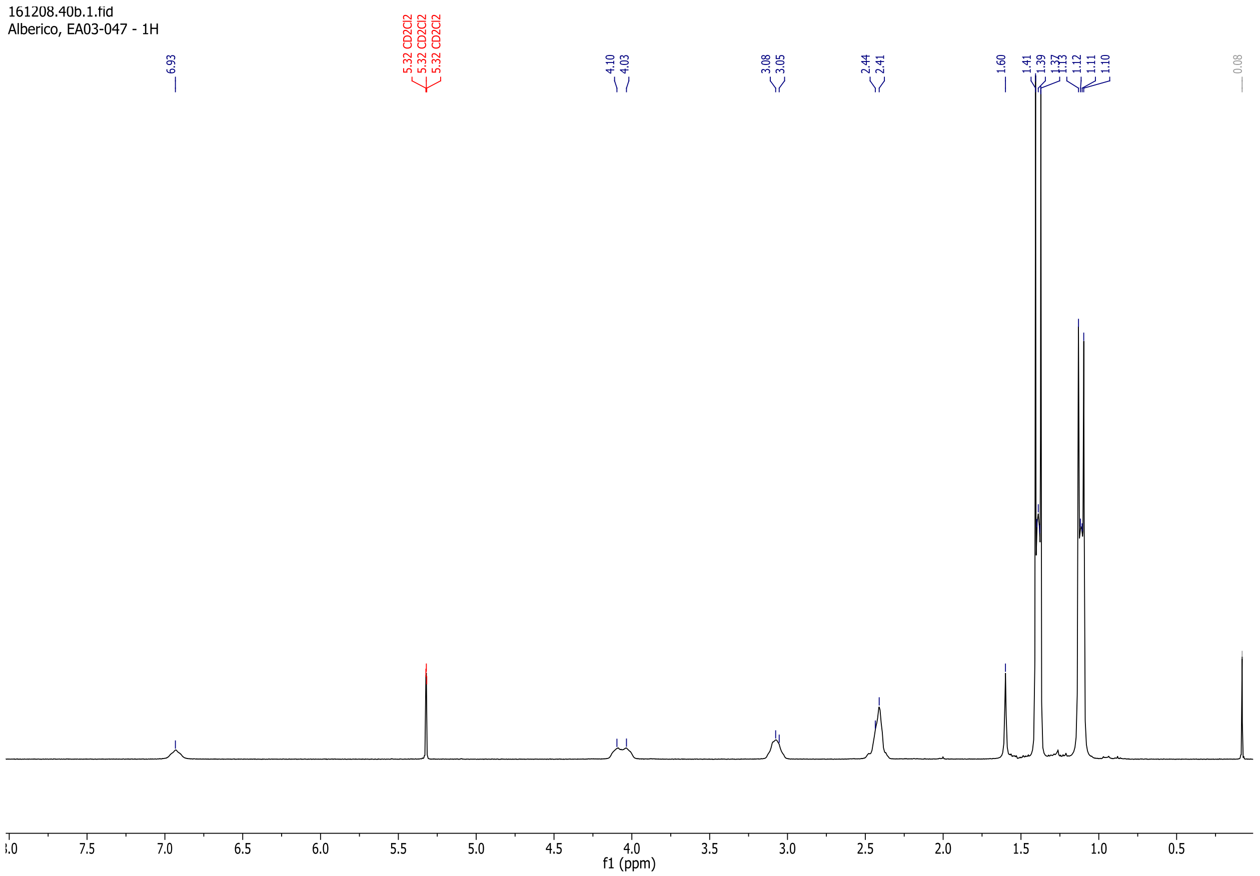

Figure S11. ^{1}H NMR (400 MHz, CD_2Cl_2, 298 K) of **Mn-3**

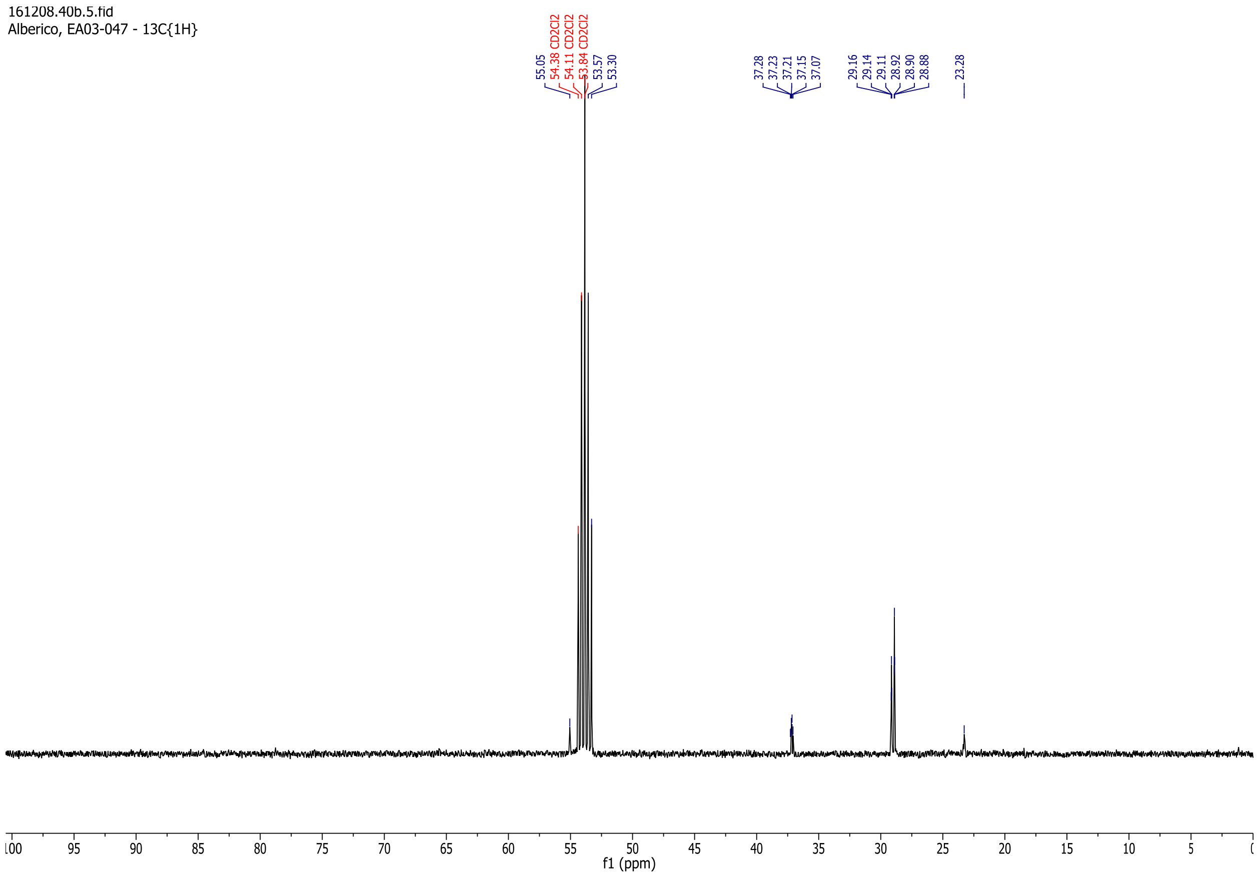

Figure S12. ^{13}C NMR (101 MHz, CD_2Cl_2, 298 K) of **Mn-3**

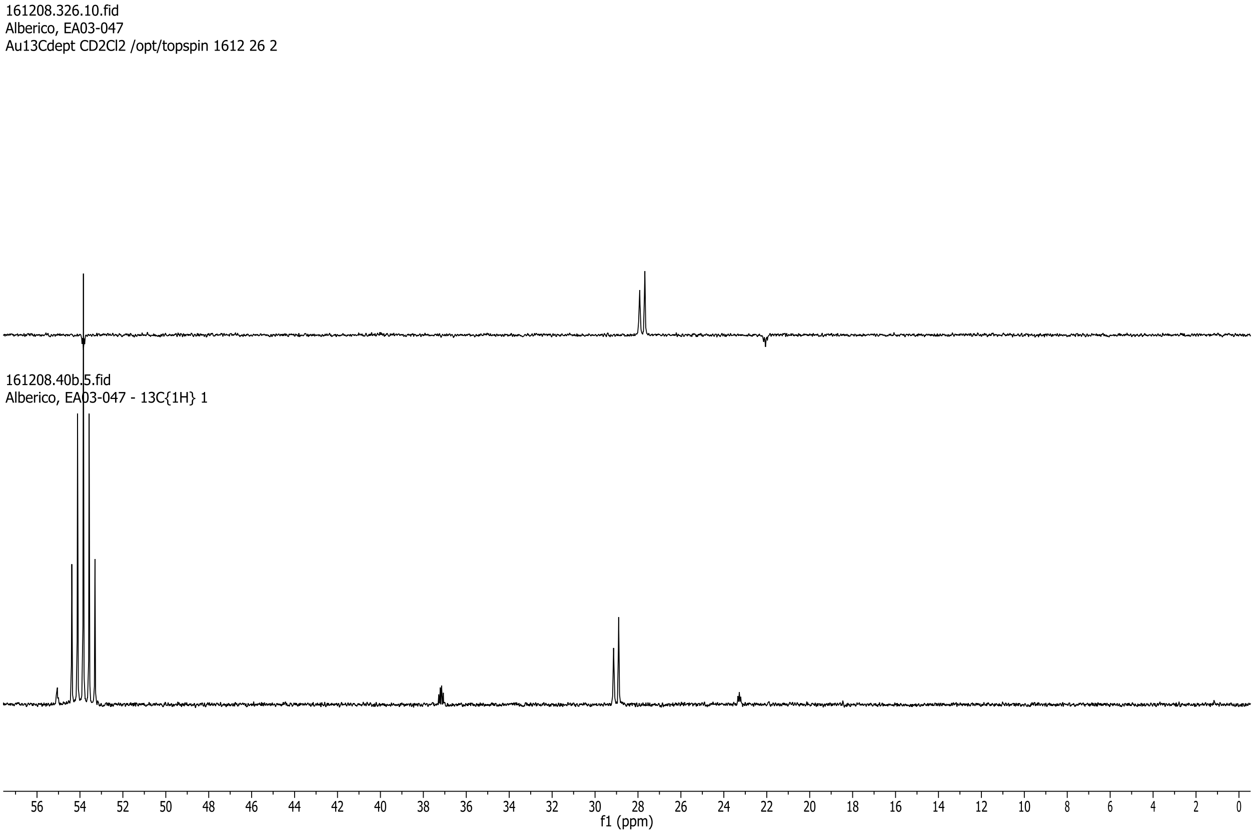

Figure S13. ^{13}C NMR (bottom spectrum) and DEPT (upper spectrum) (400 MHz, CD_2Cl_2, 298 K) of **Mn-3**

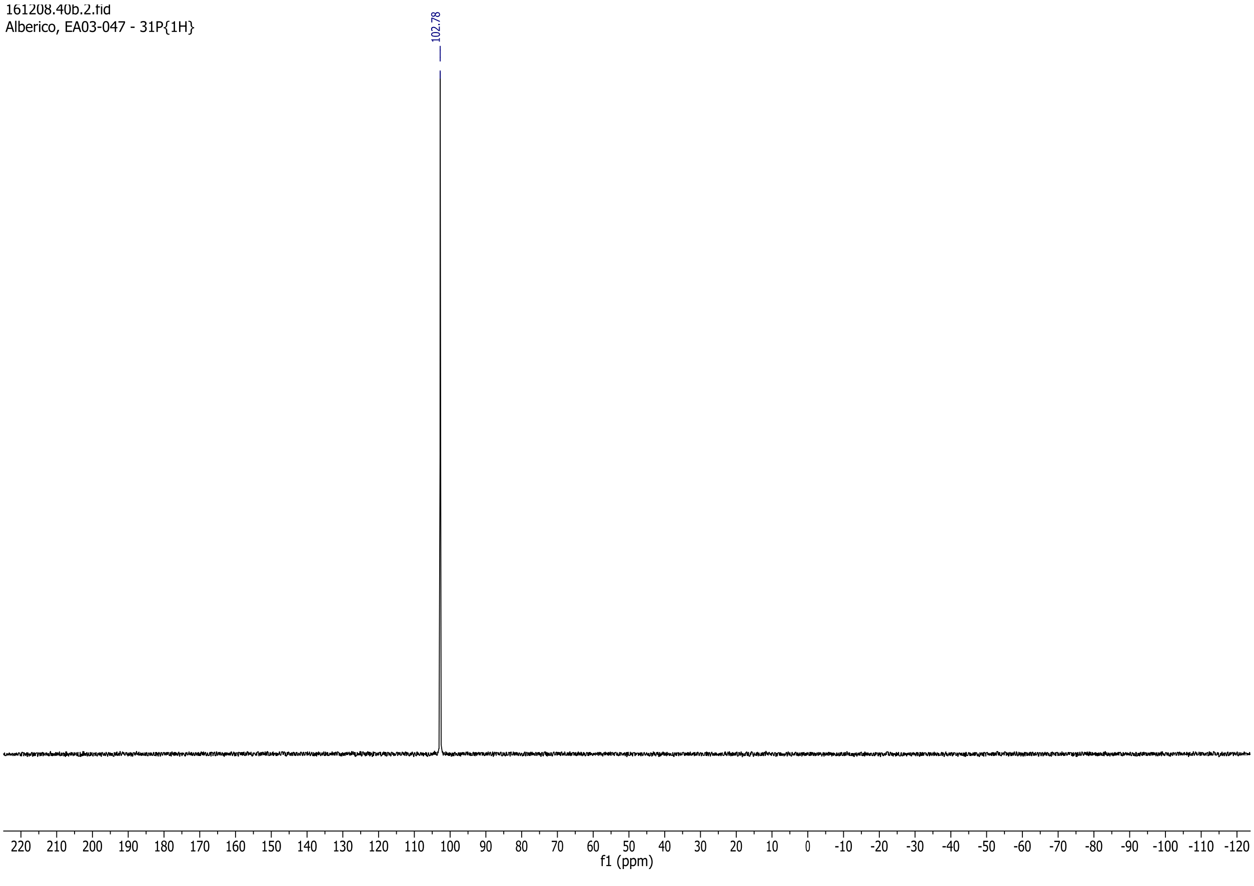

Figure S14. ^{31}P NMR (400 MHz, CD_2Cl_2, 298 K) of **Mn-3**

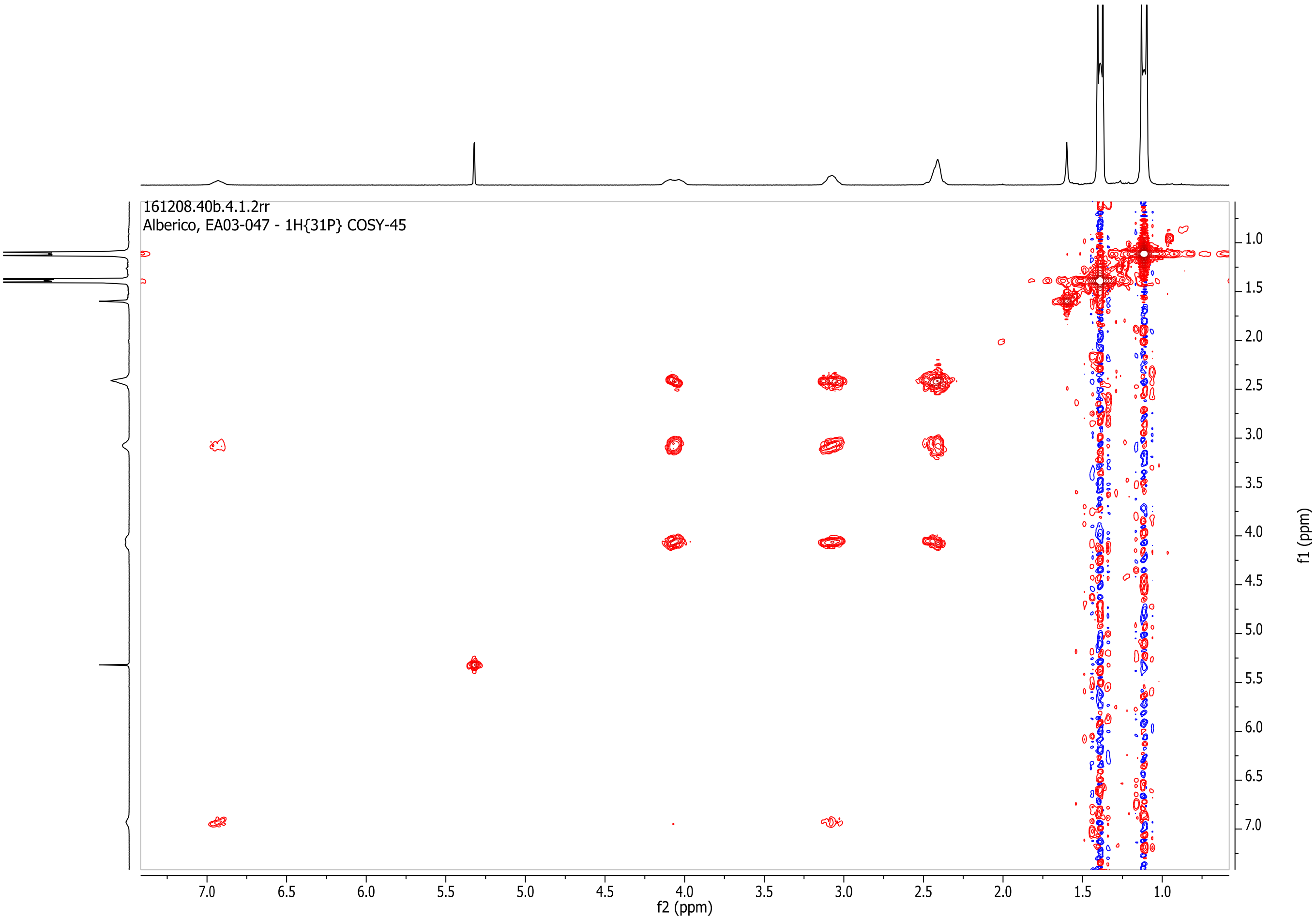

Figure S15. COSY NMR (400 MHz, CD_2Cl_2, 298 K) of **Mn-3**

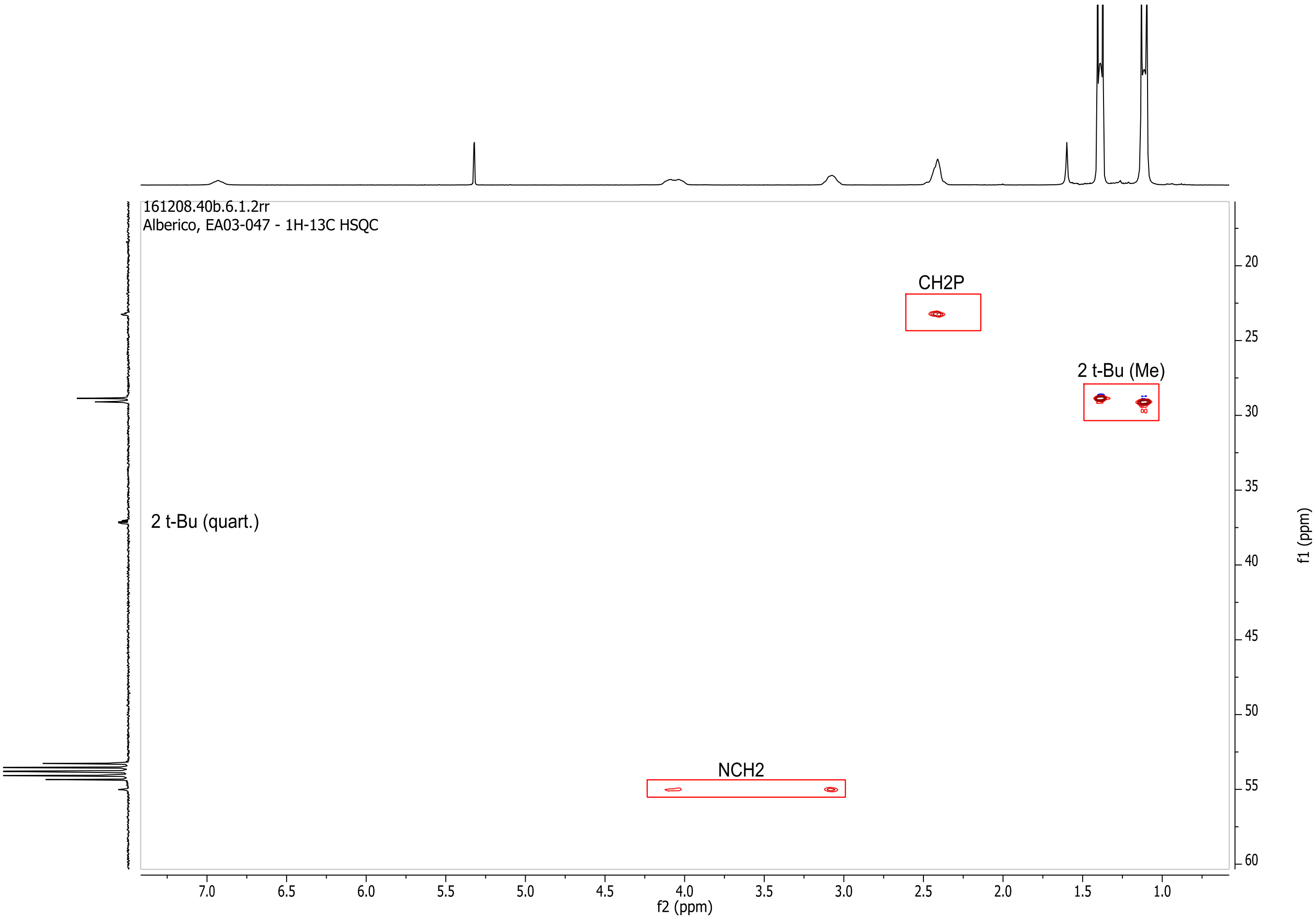

Figure S16. HSQC NMR (400 MHz, CD_2Cl_2, 298 K) of **Mn-3**

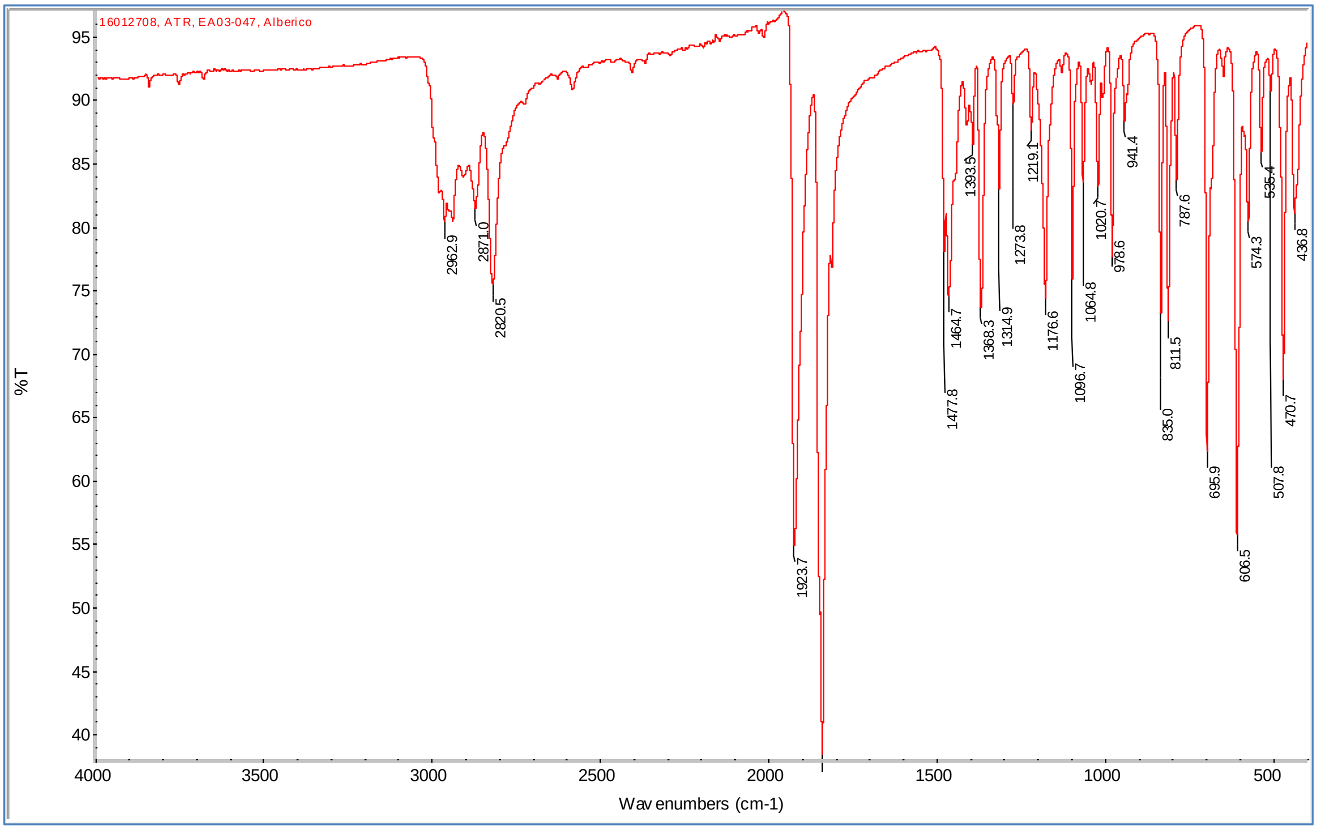

Figure S17. IR-ATR spectrum **Mn-3**.

3. GENERAL PROCEDURE FOR THE HYDROGENATION OF AMIDES

General procedure for the hydrogenation of amides: A 4 mL glass vial containing a stirring bar was sequentially charged with the amide (0.25 mmol) and complex Mn-2 (1-5 mol%). Afterwards, the reaction vial was capped with a septum equipped with a syringe needle and set in the alloy plate and the vial was purged with 3 cycles of vacuum and argon. Cyclohexane or cyclohexane / t-amyl alcohol (1.5 / 0.5) mixture (2mL) and the corresponding catalytic amount of KO^tBu were sequentially added under argon and the vial was then placed into a 300 mL autoclave. Once sealed, the autoclave was purged three times with 20 bar of hydrogen, then pressurized to the desired hydrogen pressure, and placed into an aluminum block that was preheated to the desired temperature (80–140 °C). After the desired reaction time (16–24 h, the autoclave was cooled in an ice bath and the remaining gas was carefully released. Finally, n-hexadecane (20 mg) was added as an external standard, and the reaction mixture was diluted with ethyl acetate and analyzed by gas chromatography.

4. ADDITIONAL SCHEME

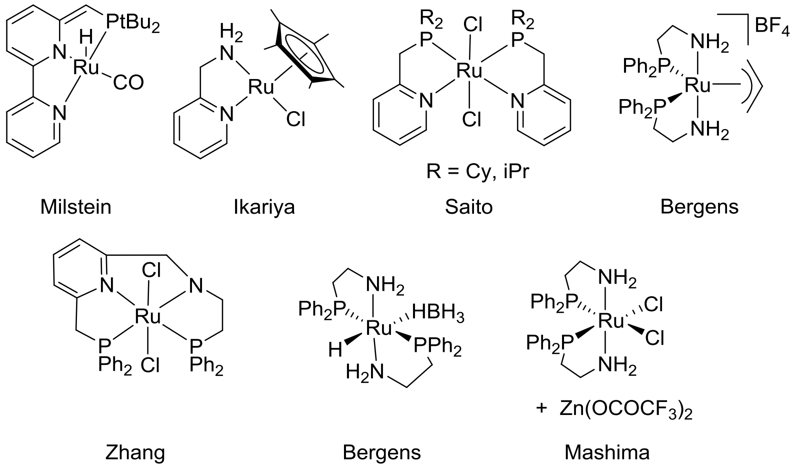

Scheme S1. Previous reported bifunctional Ruthenium catalysts that promote the hydrogenation of amides to amines and alcohols

5. REFERENCES

1. Specification of the Hg-vapor light-source: Lumatec Superlite 400, 120 W, Hg-high pressure lamp. http://www.lumatec.de/forensiclightsources/e s400.htm

2. Sheldrick, G. M., *Acta Cryst.* **2008**, *A64*, 112-122.

3. Sheldrick, G. M., *Acta Cryst.* **2015**, *C71*, 3-8.

4. R. Adam, E. Alberico, W. Baumann, H. J. Drexler, R. Jackstell, H. Junge and M. Beller, *Chem. Eur. J.,* **2016**, 22, 4991-5002.
